# Towards Sustainable Medicinal Resources through Marine Soft Coral Aquaculture: Insights into the Chemical Diversity and the Biological Potential

**DOI:** 10.3390/md20100640

**Published:** 2022-10-14

**Authors:** Ngoc Bao An Nguyen, Lo-Yun Chen, Mohamed El-Shazly, Bo-Rong Peng, Jui-Hsin Su, Ho-Cheng Wu, I-Ta Lee, Kuei-Hung Lai

**Affiliations:** 1 Graduate Institute of Pharmacognosy, College of Pharmacy, Taipei Medical University, Taipei 11031, Taiwan; 2 Department of Pharmacognosy, Faculty of Pharmacy, Ain-Shams University, Organization of African Unity Street, Abassia, Cairo 11566, Egypt; 3 National Museum of Marine Biology & Aquarium, Pingtung 94450, Taiwan; 4 Department of Marine Biotechnology and Resources, National Sun Yat-sen University, Kaohsiung 80424, Taiwan; 5 School of Dentistry, College of Oral Medicine, Taipei Medical University, Taipei 11031, Taiwan; 6 Ph.D. Program in Clinical Drug Development of Herbal Medicine, College of Pharmacy, Taipei Medical University, Taipei 11031, Taiwan; 7 Traditional Herbal Medicine Research Center, Taipei Medical University Hospital, Taipei 11031, Taiwan

**Keywords:** medicinal resource, cultured soft corals, secondary metabolites, biological functions, drug developments

## Abstract

In recent decades, aquaculture techniques for soft corals have made remarkable progress in terms of conditions and productivity. Researchers have been able to obtain larger quantities of soft corals, thus larger quantities of biologically active metabolites, allowing them to study their biological activity in many pharmacological assays and even produce sufficient quantities for clinical trials. In this review, we summarize 201 secondary metabolites that have been identified from cultured soft corals in the era from 2002 to September 2022. Various types of diterpenes (eunicellins, cembranes, spatanes, norcembranes, briaranes, and aquarianes), as well as biscembranes, sterols, and quinones were discovered and subjected to bioactivity investigations in 53 different studies. We also introduce a more in-depth discussion of the potential biological effects (anti-cancer, anti-inflammatory, and anti-microbial) and the mechanisms of action of the identified secondary metabolites. We hope this review will shed light on the untapped potential applications of aquaculture to produce valuable secondary metabolites to tackle current and emerging health conditions.

## 1. Aquaculture Background

Nature has contributed to all aspects of drug discovery and development through the production of secondary metabolites with diverse chemical structures and biological activities. In addition to terrestrial plants and animals, marine organisms are considered a prolific resource of highly potent compounds. Although occupying 70% of the earth surface, only less than 5% of deep-sea organisms have been studied [1], which opens an enormous opportunity for marine drug discovery, especially with the assistance of newly developed specialized techniques and equipment. Among marine organisms, soft corals are marine animals that belong to the order Alcyonacea. Thousands of secondary metabolites have been isolated from soft corals with a wide range of biological activities, such as antibacterial, antitumor, anti-inflammatory, antifungal, antiviral, antioxidant, antiallergic, and antimalarial activities [2]. However, the interesting compounds are usually extracted in very low amounts. Once a compound is isolated and identified as a potent therapeutic agent by in vitro or in vivo assays, a kilogram-scale amount could be required for further clinical investigations and even for commercial supply. The need for these large amounts urges the development of large-scale and sustainable techniques to maintain a consistent supply of potent marine secondary metabolites for drug development. 

Total synthesis and semisynthesis techniques are some possible options for the consistent supply of these marine-derived products. However, these approaches may face several difficulties due to the need for the accurate identification of the complex structures of the extracted compounds. Despite the recent advances in structural elucidation techniques, there are misassignments of the newly identified compounds, sometimes with the formula, constitution, configuration of double bonds, absolute configuration, and one or several stereocenters [3]. This hindered the development of synthetic or semisynthetic routes from producing complex marine natural products. 

Aquaculture emerged as an excellent choice to meet the high demands for consistent and sustainable resources of potential marine-derived natural products. Coral culture started in the late 1950s mainly for commercial and reservation purposes in many regions, such as Japan, the Philippines, Central Pacific, South Pacific, North America, Sri Lanka, Australia, Taiwan, and Thailand [4]. Since then, many techniques have been exploited to facilitate the mass production of soft corals and to expand the diversity of cultured corals by direct transplantation, coral gardening, micro-fragmentation, and larval enhancement [5]. Some of these methods can be deployed ex situ and in situ [5]. Although in situ approach is simpler and cheaper than ex situ method [6]. However, ex situ is more suitable and preferable to be employed for the pharmaceutical supply of soft corals because the culture conditions are controllable and reproducible. The compounds of interest can be obtained consistently by maintaining stable environmental conditions in the culture system. The optimization of critical parameters can be performed to maximize the metabolite production of the soft corals [6]. The chemical diversity of secondary metabolites derived from cultured soft corals can be increased by adjusting the environmental conditions involving temperature, salinity, nutrient concentrations, and turbidity [2]. 

The soft corals are collected from the wild and cultured in a tank for several years before harvesting and extraction. Few studies reported the specific parameters of the culture systems that were used to grow the soft corals for drug discovery. The aquaria or cultivation tanks, which are the main part of the culture system, are usually equipped with coral sand; artificial rocks; artificial corals reefs; live sea sand; live sea rocks; marine creatures, such as fishes, snails, sea urchins, and sea cucumbers; and other cultured soft corals [7,8,9]. Seawater is used as the main medium for the cultural environment of soft corals. The salinity of seawater is also controlled at about 36 practical salinity units (psu) [9]. The temperature is maintained between 24 °C and 28 °C by a cooler. LED light is utilized to mimic the sunshine, and a foam fractionator is also used to remove waste particles from the system [7,8,9].

The diverse chemical constituents of soft corals and their highly potent bioactivities have attracted great interest from scientists worldwide, resulting in a huge quantity of studies on these marine organisms. Due to the vast number of related research, there are demands for general overviews and brief summaries of these studies. As a result, numerous reviews concerning the natural products of soft corals have been published over the past decades with different aims. Half of the review publications focused on the chemical diversity of specific genera, such as *Xenia* [10], *Cespitularia* [11], *Alcyonium* [12], *Sacrophyton* [13], *Sinularia* [14,15], and *Lobophytum* [14], or on a particular species, e.g., *Sinularia flexibilis* [16,17]. Several reviews showed their interests in a limited type of secondary metabolites—mainly cembranoids [14], steroids [18,19], and terpenoids [10,12,14,20]—or in compounds exhibiting certain bioactivities, such as anti-inflammatory metabolites [21,22]. Overall, the similarity of these publications was that the soft corals were mentioned in general, whether they were wild types or were harvested from cultivation. This is the biggest difference between our current work and the previous reviews. 

**Figure 1:**
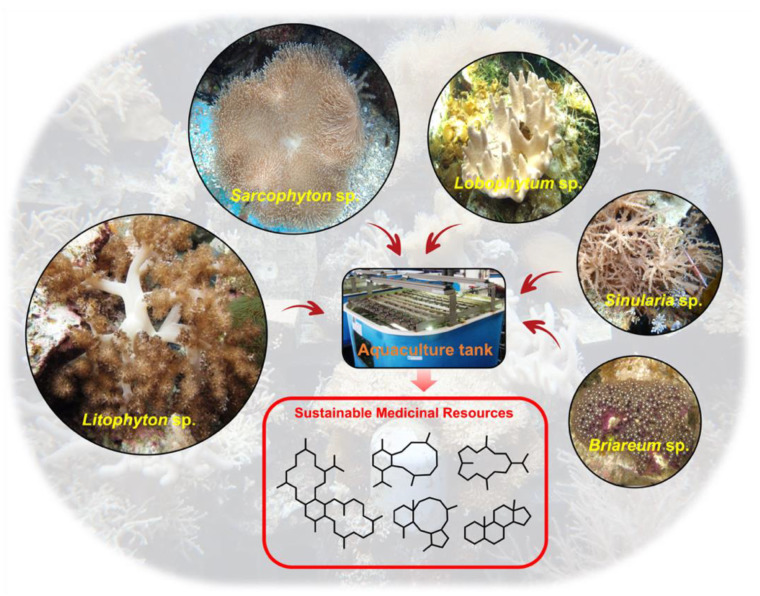
Selected cultured soft corals with rich medicinal resources.

In this review, we aim to provide a general summary of the chemical diversity of natural products derived from cultured soft corals and their potential bioactivities (Figure 1). Different keywords, such as “cultured”, “soft corals”, and “secondary metabolites”, were searched in various platforms, including Pubmed, Google Scholar, ScienceDirect, ResearchGate, and Reaxys. A total of 86 references with 201 derivatives were found to be related to the target topic and discussed throughout the current review. We try in this work to highlight that the aquaculture of these marine organisms can provide a consistent, reproducible, and sustainable source of potent marine compounds, aiding in the discovery and development of new drugs from both known and novel bioactive secondary metabolites.

## 2. Secondary Metabolites Derived from Cultured Soft Corals

### 2.1. Diterpenes 

Diterpenes were the most common compounds discovered from cultured soft corals. They showed the most diverse structural variations with six subclasses, including eunicellin-based diterpene, cembrane-type diterpene, spatane-type diterpene, norcembranoidal diterpene, briarane-type diterpene, and aquariane-type diterpene.

#### 2.1.1. Eunicellin-Based Diterpene

**Figure 2:**
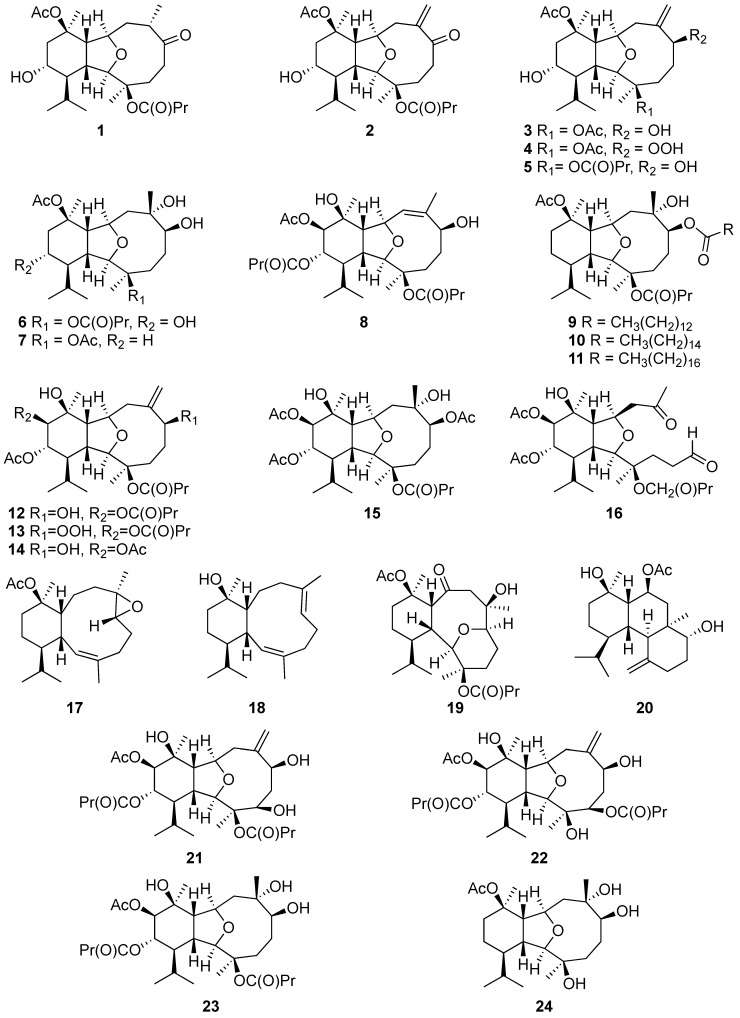
Eunicellin-based diterpenes (**1**–**24**) were identified from cultured soft corals.

**Figure 3:**
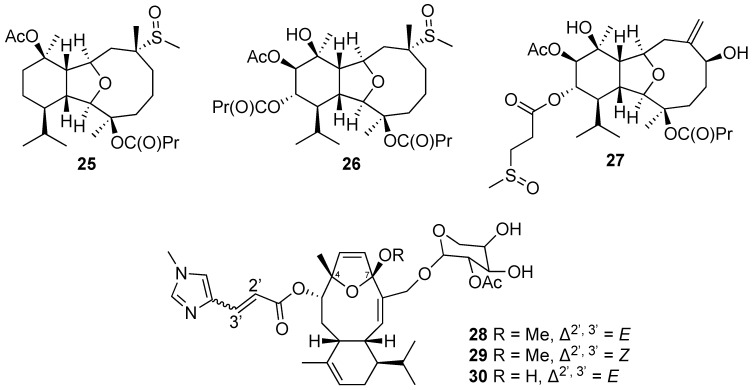
Sulfur-containing diterpenes (**25**–**27**) and diterpene glycosides (**28**–**30**) were identified from the cultured soft corals.

Eunicellin-based diterpenes (Figure 2 and Figure 3) are marine natural products that consist of a six-membered ring moiety fused to a ten-membered ring moiety. In many compounds, an additional ether bridge is formed between C-2 and C-9 or C-4 and C-7 [23].

The investigation of new compounds from the cultured soft coral *Klyxum simplex* led to the isolation and identification of 27 novel secondary metabolites, including klysimplexins A–X (compounds **1**–**24**) [24,25,26] and klysimplexin sulfoxides A–C (compounds **25**–**27**) [27]. Most of the compounds were obtained as a colorless oil, except for klysimplexins A, which consisted of colorless crystals following recrystallization from acetone. Their molecular formulas were established based on their HRESIMS spectra and ^13^C NMR data. The IR spectra of klysimplexin sulfoxides showed that they not only possess hydroxy, carbonyl, and ester functionalities as revealed in the spectra of klysimplexins but also contain sulfoxide groups in their structure. In general, these compounds share a common eunicellin-based skeleton which was partially elucidated with the assistance of IR, 1D, and 2D NMR spectroscopic data. Single-crystal X-ray diffraction analysis was exploited to establish the detailed structure and relative configuration of klysimplexin A (**1**). The absolute configurations of klysimplexins A (**1**), C (**3**), L (**12**), and V (**22**) were further determined by the modified Mosher’s method [24,25,26]. The NMR spectroscopic data confirmed that the eunicelline-based structure of klysimplexin I (**9**) was similar to a known compound, simplexin B, which was isolated from the same species. The replacement of a hydroxy group at C-6 in simplexin B by a myristate moiety at C-6 in 9 was confirmed by a base-catalyzed hydrolysis of **9**, which afforded simplexin B after the reaction [25]. The authors indicated that klysimplexin P (**15**) is a 6,7-secoeunicellin while klysimplexin T (**20**) possesses a tricarbocyclic skeleton, which might be derived from the carbon–carbon bond formation between C-2 and C-7 of the corresponding 2,9-deoxygenated eunicellin. Among these novel metabolites, klysimplexins U (**21**) and V (**22**) were reported as the first 4-oxygenated eunicellin-based diterpenes that were isolated from the genera *Klyxum* [26], and klysimplexin sulfoxides A–C (compounds **25**–**27**) were the first discovered sulfoxide-containing eunicellin-type derivatives [27] (Figure 3).

Three diterpene glycosides, including eleutherobin (**28**), (*Z*)-eleutherobin (**29**), and desmethyleleutherobin (**30**), were reported from the cultured *Erythropodium caribaeorum*. The aglycone moieties of these glycosides share a common eunicellane-based skeleton that contains an oxygen bridge between C-4 and C-7 in the ten-membered ring [28] (Figure 3).

#### 2.1.2. Cembrane-Type Diterpene

**Figure 4:**
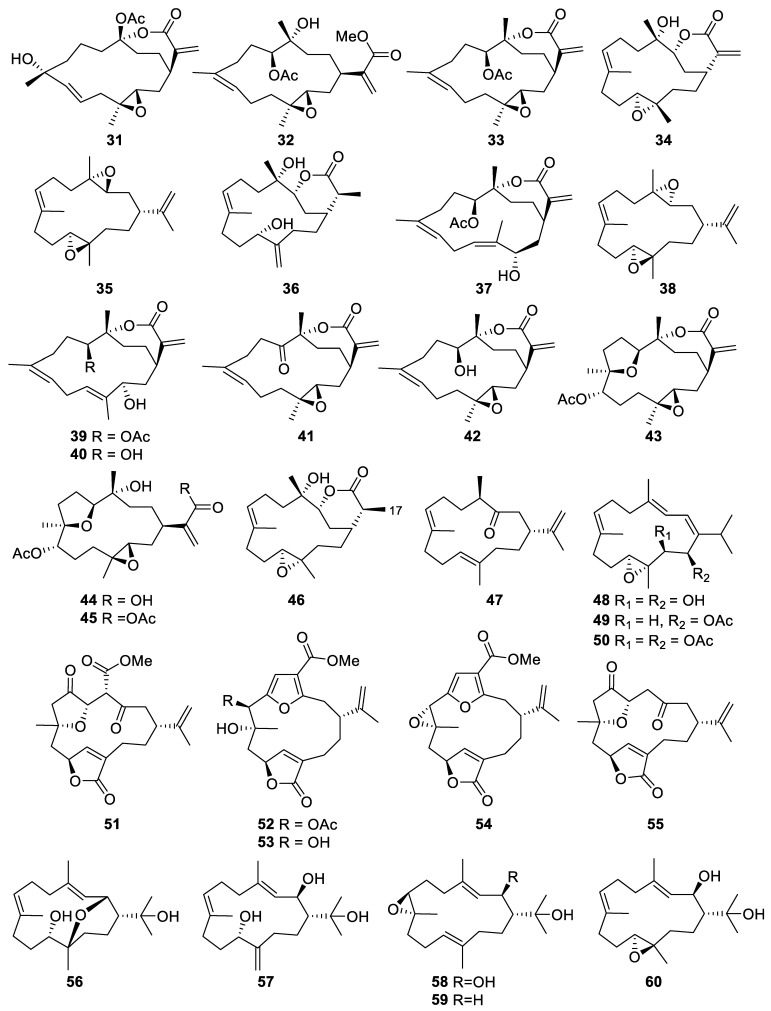
Cembranoids (**31**–**60**) were isolated from the aquaculture soft corals of *Sinularia* and *Litophyton* genera.

**Figure 5:**
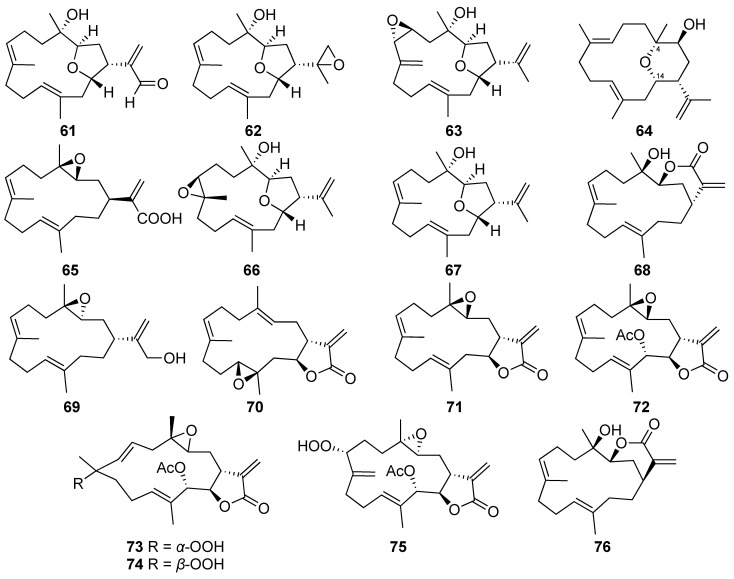
Cembranoids (**61**–**76**) isolated from the aquaculture soft corals of *Lobophytum* and *Sarcophyton* genera.

**Figure 6:**
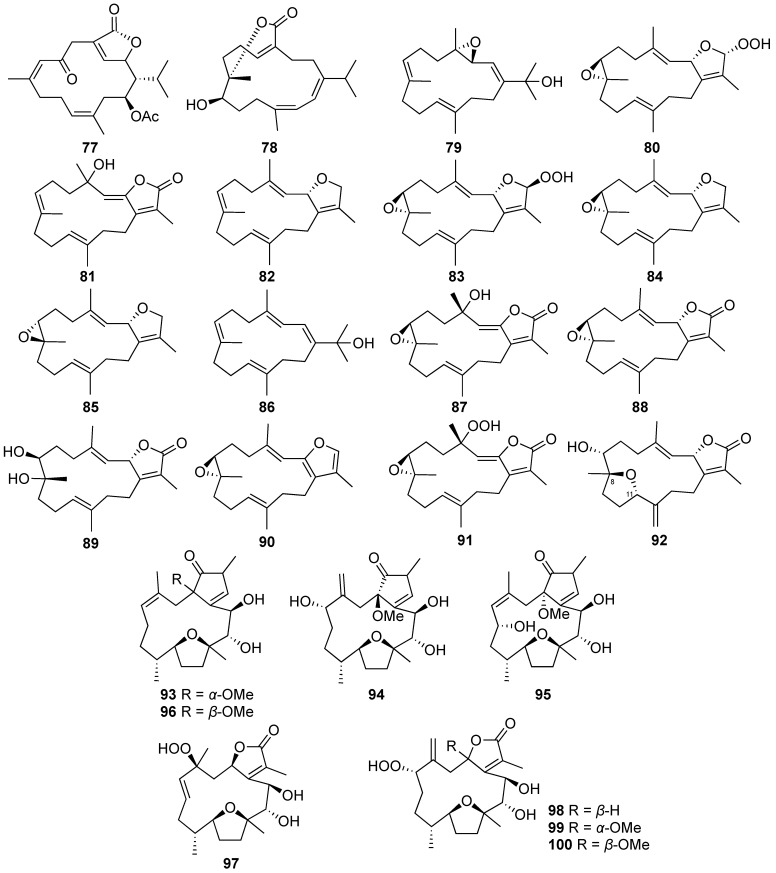
Cembranoids (**77**–**100**) isolated from the aquaculture soft corals of *Sarcophyton* and *Briareum* genera.

Cembrane-type diterpenes or cembranoids (Figure 4, Figure 5 and Figure 6) are a large group of natural products that possess a 14-member carbocyclic ring derived from the cyclization of geranylgeranyl pyrophosphate and carry diverse functional groups, such as lactone, epoxide, furan, ester, aldehyde, hydroxyl, and carboxyl moieties [14].

An investigation of the chemical constituents of the cultured soft coral *Sinularia flexibilis* led to the purification of three cembranoids, including flexibilisolide A (**31**), flexibilisin A (**32**), and 11-*epi*-sinulariolide acetate (**33**) [29]. 11-*epi*-Sinulariolide acetate was a known compound that was originally isolated and identified from the wild soft corals *Sinularia notanda* and *Sinularia querciformis* collected from the Gulf of Elat [30]. The comparison of NMR data of **32** with those of **33** showed a similarity in the structure, except for an additional methoxy group that was observed in **32**. This was confirmed by a base-catalyzed hydrolysis of **33** that afforded **32** as a product of the reaction [29].

Flexibilide (**34**), also known as sinularin, was first isolated from the soft coral *Sinularia flexibilis* collected from the Hayman Island of Australia. Its structure was established by NMR and X-ray crystallography. The anti-inflammatory and analgesic effects of flexibilide were performed using a material isolated from the same species that was cultured in a cultivation tank of the National Museum of Marine Biology and Aquarium in Taiwan. The purity of flexibilide was identified and verified by ^1^H and ^13^C NMR spectra before the in vivo study [31].

Three new cembranoids, diepoxycembrene B (**35**), dihydromanaarenolide I (**36**), and isosinulaflexiolide K (**37**), along with eight known isolates, including diepoxycembrene A (**38**), sinulaflexiolide K (**39**), (−)-sandensolide (**40**), 11-dehydrosinulariolide (**41**), sinulariolide (**42**), 3,4:8,11-bisepoxy-7-acetoxycembra-15(17)-en-1,12-olide (**43**), dendronpholide F (**44**), and dendronpholide G (**45**), were collected from the cultured soft coral *Sinularia flexibilis*. The structures and absolute stereochemistry of **37** and **44** were confirmed by single-crystal X-ray diffraction [32].

Dihydrosinularin (**46**), a cembranoid derivative, was also obtained from the cultured *Sinularia flexibilis*. The compound was further tested for its anti-inflammatory activity and showed no inhibitory effects on the NO expression due to a slight difference with active derivative. Hydrogenation at C-17 formed a single bond in **46** instead of a double bond as in sinularin [33].

The ethyl acetate extract of the cultured soft coral *Sinularia gibberosa* afforded new cembrane-based diterpenes, cugibberosene A (**47**), along with three known metabolites, 11,12-epoxy-13,14-dihydroxycembrene-C (**48**), flaccidoxide (**49**), and flaccidoxide-13-acetate (**50**) [34].

Two new cembranes, 4-carbomethoxyl-10-*epi*-gyrosanoldie E (**51**) and 7-acetylsinumaximol B (**52**), along with three known cembranoid derivatives, were isolated from the cultured *Sinularia sandensis*. Other known isolates were identified as sinumaximol B (**53**), pukalide (**54**), and 10-*epi*-gyrosanoldie E (**55**), based on a comparison of their spectroscopic data with the published spectra. The absolute configuration of **54** was also confirmed by single-crystal X-ray diffraction analysis [32].

Two new metabolites, columnariols A (**56**) and B (**57**) were isolated from the cultured soft coral *Litophyton columnaris* as a colorless oil [35]. Additionally, three novel compounds were obtained from the organic extract of the same species, including 2β-hydroxy-7β,8α-epoxynephthenol (**58**), 2β-hydroxy-11α,12β-epoxynephthenol (**59**), and epoxynephthenol (**60**) [36].

The investigation of secondary metabolites from the cultured soft coral *Lobophytum crassum* led to the discovery of five new cembranes, culobophylins A-E (**61**–**65**), along previously reported cembranoids, including lobophylins A (**66**) and B (**67**), lobocrassins B (**68**) and C (**69**), crassocolide E (**70**), sarcocrassolide (**71**), 13-acetoxysarcocrassolide (**72**), sarcocrassocolides F (**73**), G (**74**) and M (**75**), and 14-deoxycrassin (**76**). The studies revealed that culobophylin B (**59**) was a rare cembranoid possessing an isopropyl moiety with an epoxide group, while culobophylin D (**64**) was the first cembranoid exhibiting saturated internal C4-O-C14 linkage five-membered ring among all cembrane-type diterpenes [7,37].

A biogenic dienophile precursor of biscembranoid, isosarcophytonolide D (**77**), was isolated from the cultured soft coral *Sarcophyton glaucum* [38]. Along with another formerly reported compound, (4*Z*,8*S*,9*S*,12*Z*,14*E*)-9-hydroxy-1-isopropyl-8,12-dimethyloxabicyclo [9.3.2]-hexadeca-4,12,14-trien-18-one (**78**), compound **77** was also isolated and identified from the cultured *Sarcophyton digitatum* [8].

The chemical examination of the cultured soft coral *Sarcophyton tenuispiculatum* led to the discovery of three new cembranoids, sarcotenusenes A-C (**79**–**81**), and ten previously reported metabolites, sarcophytonins A (**82**) and F (**83**), (2*S*,7*S*,8*S*)-sarcophytoxide (**84**), (2*S*,7*R*,8*R*)-sarcophytoxide (**85**), dehydronephthenol (**86**), 3,4-dihydro-4α-hydroxy-∆^2^-sarcophine (**87**), (+)-sarcophine (**88**), (+)-7α,8β-dihydroxydeepoxysarcophine (**89**), 2,16:7*S*,8*S*-diepoxy-1,3,11,15-cembratetraene (**90**), and a hydroperoxide obtained by the autoxidation of dihydrofuranocembranoid (**91**) [9]. 

A new cembranoid, trocheliophorol A (**92**), was isolated from the cultured soft coral *Sarcophyton trocheliophorum*. Based on the findings from an extensive analysis of the spectral data of **92**, it was demonstrated that the novel compound was obtained as the first example of an α,β-unsaturated-γ-lactone cembrane possessing a tetrahydrofuran moiety with a rare 8,11-ether linkage [39]. 

Three new furanocembranoids-briaviodiol F (**93**) and briaviotriols A (**94**) and B (**95**), along with a known analogue, briaviodiol A (**96**), were discovered from the cultured-type *Briareum violaceum*. The structures of the novel compounds were determined as tricyclic cembrane diterpenes possessing tetrahydrofuran rings by the interpretation of their spectroscopic data. The known metabolite **96** was identified as briaviodiol A through the comparison of its ^1^H and ^13^C NMR spectra with the published data [40]. 

A NMR-directed investigation of chemical constituents from the cultured octocoral *Briareum violaceum* led to the discovery of four new hydroperoxyfurancembranoids, briaviodiols B–E (**97**–**100**) [41].

#### 2.1.3. Spatane-Type Diterpene

Spatane-type diterpenes are tricyclic terpenoids derived from a prenylgermacrane by 1,5- and 6,10-cyclisation [42].

**Figure 7:**
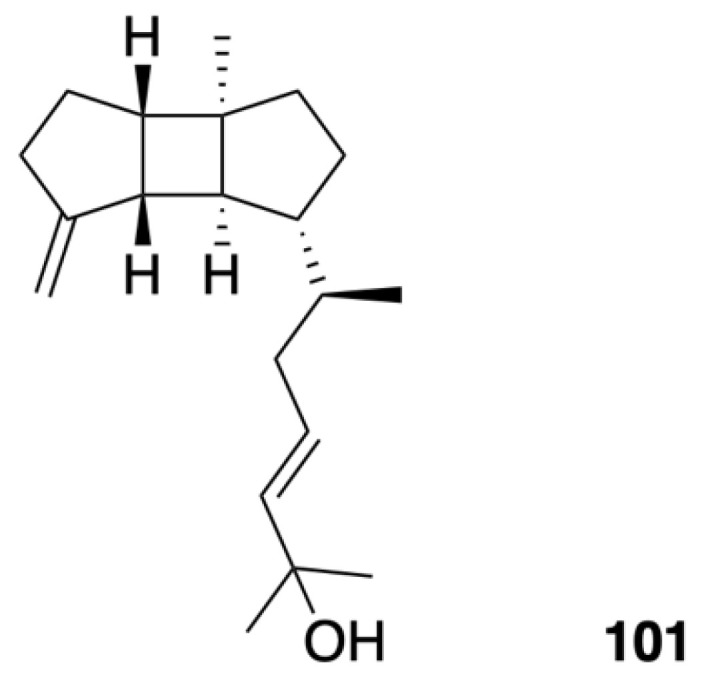
A spatane-type diterpene, leptoclalin A (**101**).

Leptoclalin A (**101**) (Figure 7) was isolated as a colorless oil from cultured soft coral *Sinularia leptoclados*. Its molecular formula was determined as C_20_H_32_O based on its HRESIMS spectrum. The detailed analysis of its spectroscopic data, in particular 2D NMR, resulted in the establishment of a new rare spatane diterpenoid [43].

#### 2.1.4. Norcembranoidal Diterpene

**Figure 8:**
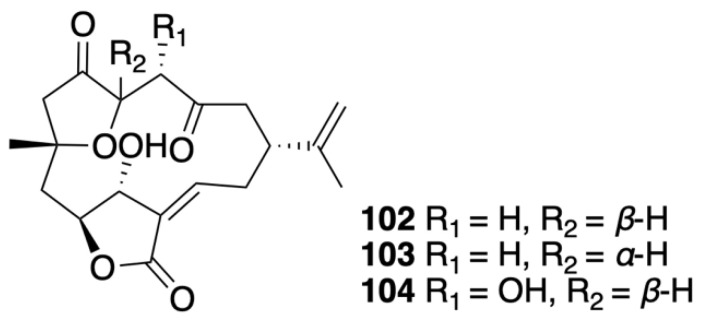
Norcembranoids (**102**–**104**) were isolated from the cultured *Sinularia numerosa*.

Norcembranoids are diterpene derivatives that lack a C-18 carbon substituent in comparison with C_20_-cembranoids [44]. Several norcembranoidal diterpenes were discovered from the culture of soft corals (Figure 8). For instance, an investigation of the chemical constituents of *Sinularia leptoclados* led to the isolation of two known norcembranoid diterpenes, 5-*epi*-sinuleptolide (**102**) and sinuleptolide (**103**) [43]. 5-*epi*-Sinuleptolide, along with a new analogue, 4α-hydroxy-5-*epi*-sinuleptolide (**104**), was also purified and identified from cultured soft coral *Sinularia numerosa* [45].

#### 2.1.5. Briarane-Type Diterpene

**Figure 9:**
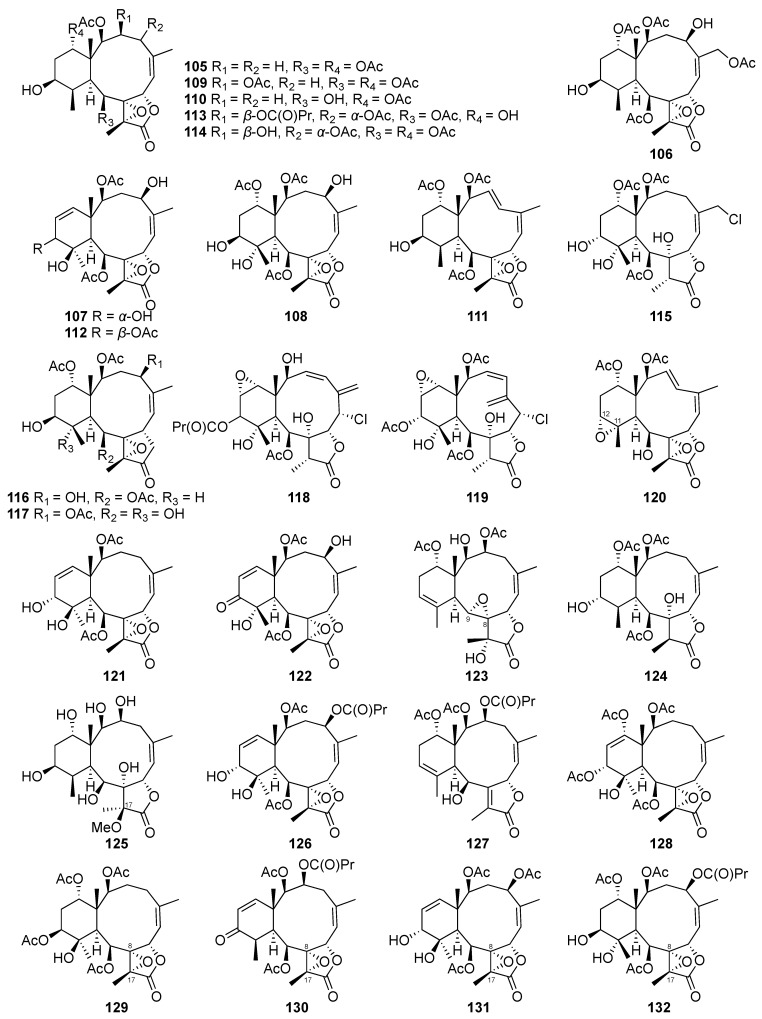
Briarane-type diterpenes (**105**–**132**) were isolated from the cultured *Briareum stechei*.

**Figure 10:**
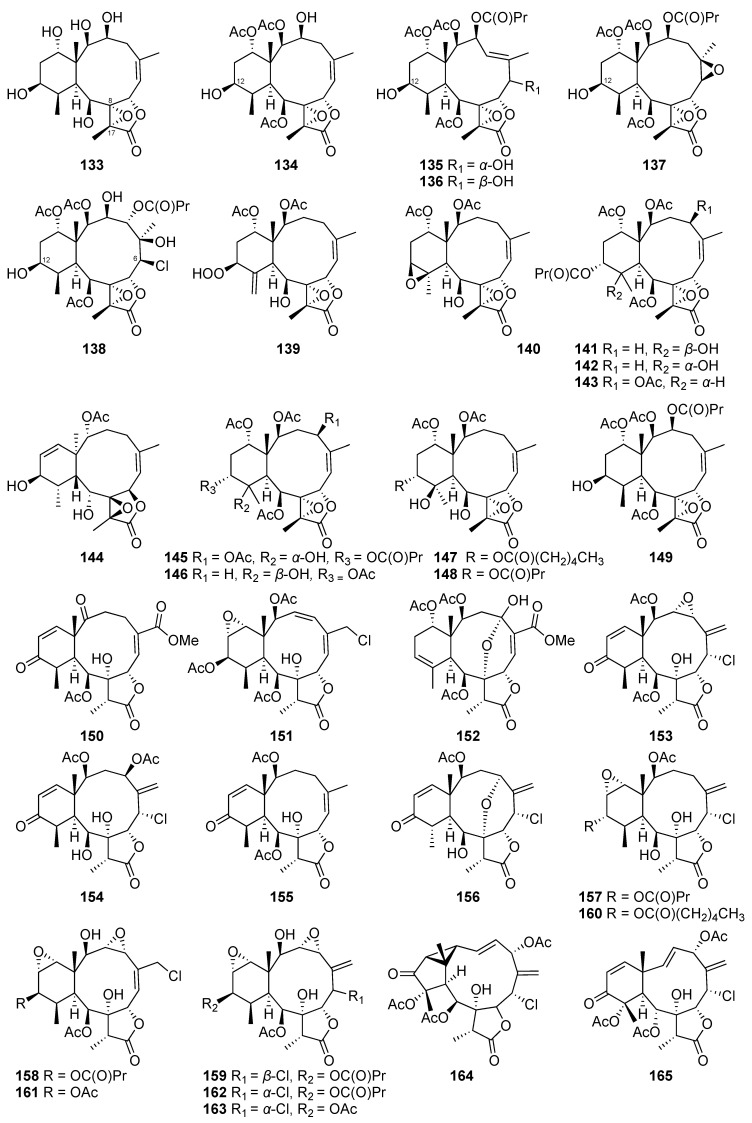
Briarane-type diterpenes (**133**–**165**) were isolated from the cultured *Briareum* sp. and *Erythropodium caribaeorum*.

**Figure 11:**
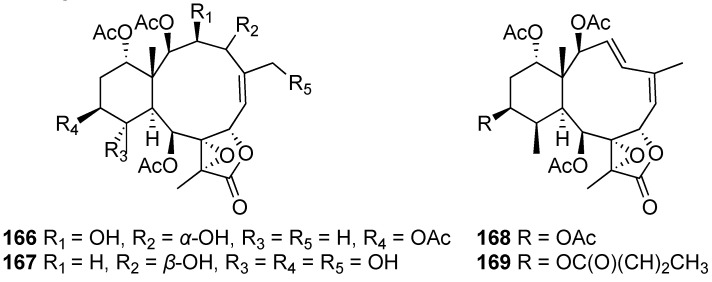
Newly isolated briaranes from the cultured soft coral *Briareum violaceum* (**166**–**169**).

Briarane-type diterpenes (Figure 9, Figure 10 and Figure 11) are derived from the 3,8-cyclization of cembranoid [46]. This type of diterpenoid skeleton is only found in marine organisms, particularly in octocorals [47]. The chemical investigation of the cultured scleraxonia *Briareum stechei* led to the discovery of four new diterpenoids-briaexcavatins I–L (**105**–**108**). Other two known compounds, excavatolides C (**109**) and E (**110**), were first found in the wild-type *Briareum stechei* discovered in Taiwan and were also isolated from the culture-type coral. The absolute configurations of **109** and **110** were further confirmed by X-ray data analysis for the first time [48].

Guided by the proton NMR signals for interesting structures, the ethyl acetate layer that was obtained by partitioning the organic extract of the cultured *Briareum stechei* afforded four new briarane-related diterpenes. They were assigned as briaexcavatins M–P (**111**–**114**) based on the analysis of extensive spectroscopic data [49]. Subsequently, briaexcavatins Q–T (**115**–**118**) were isolated from the same cultured species [50]. 

A new chlorinated briarane, briaexcavatin U (**119**), was also isolated from a cultured octocoral *Briareum stechei*. After isolation as a white powder, the compound was recrystallized as colorless prisms for further X-ray analysis to establish its absolute stereochemistry [51].

Five new briarane derivatives, briaexcavatins V–Z (**120**–**124**), were isolated from the cultured octocoral *Briareum stechei*. The absolute stereochemistry of briaexcavatin X (**121**) was confirmed on the basis of single-crystal X-ray diffraction data. In addition to the structure elucidation, the relationships between ^13^C NMR chemical shifts and the conformations of briaranes possessing an 11,12-epoxy group were also discussed. Among the five novel compounds, briaexcavatin Y (**123**) was the first example of a briarane possessing a C-8/9 epoxy group [52]. 

The long-term study of *Briareum stechei* chemical constituents led to the isolation of two novel briarane derivatives, excavatoids A (**125**) and B (**126**), and a known metabolite, briaexcavatin I (**105**). Compounds **125** and **105** were subjected to a single-crystal X-ray diffraction analysis to establish their absolute configurations. Among these isolates, excavatoid A (**125**) was reported as the first briarane, possessing six hydroxy groups and a 17-methoxy group [53].

Two new briarane-type diterpenes that were assigned as excavatoids E (**127**) and F (**128**) were isolated from the cultured octocoral *Briareum stechei* [54]. The continuing investigation for new natural products from the cultured octocoral *Briareum stechei* led to the discovery of five new 8,17-epoxybriarane diterpenoids, excavatoids G–K (**129**–**133**), and five new 12-hydroxybriarane diterpenoids, excavatoids L–P (**134**–**138**). Among those novel isolates, excavatoid P (**138**) demonstrated the first example of briarane possessing a 6β-chlorine atom in its molecular framework. The absolute stereochemistry of **129** was further confirmed by single-crystal X-ray diffraction analysis [55,56,57]. 

A new 12-hydroperoxybriarane, briarenolide D (**139**), and a known briarane, 2β-acetoxy-2-(debutyryloxy)stecholide E (**140**) were obtained from the cultured-type *Briareum* sp. The absolute configurations of **139** were established by a single-crystal X-ray diffraction analysis for the first time [47].

In the study of secondary metabolites from the cultured octocoral *Briareum violaceum*, a series of seven new briaranes, briaviolides K–Q (**141**–**147**), were discovered. Additionally, a known briarane analogue, excavatolide Z (**148**), was also obtained from the same animal material [58,59,60]. The chemical examination of the cultured octocoral *Briareum stechei* resulted in the isolation of an anti-inflammatory briarane derivative, excavatolide B (**149**) [61,62].

Two new metabolites that possess a briarane skeleton were obtained by the analysis of chemical constituents of the cultured octocoral *Briareum stechei*. The identified compounds were designated as briarenols O (**150**) and P (**151**), in which briarenol O (**150**) was believed to be the second analogue that possesses a rare 2-ketobriarane skeleton. At the same time, a known analogue, excavatolide C (**109**), was also isolated along with the novel derivatives [46].

Four new compounds, briarenols Q–T (**152**–**155**), were obtained from the organic extract of the cultured octocoral *Briareum stechei* using high performance liquid chromatographic approaches [63].

Eight chlorinated briarane diterpenoids were purified from the organic extract of the cultured-type *Briareum stechei*. The structure of four previously unreported metabolites, briarenols W-Z (**156**–**159**), were established using spectroscopic analyses, while the known ones were identified as solenolide A (**160**), briarenolide M (**161**), briaexcavatolide F (**162**), and brianolide (**163**) based on the comparison of their spectroscopic data with literature values. The absolute configuration of **163** was also determined using single-crystal X-ray diffraction analysis [64]. The methanol extract of the cultured *Erythropodium caribaeorum* afforded two briaranes, erythrolides A (**164**) and B (**165)**, which were also found in the wild type of the same species [28].

In a recent study on the chemical constituents of the cultured soft coral *Briareum violaceum*, three new polyacetoxybriarane diterpenoids, briavioids A-C (**166**–**168**), as well as two known analogues, briaexcavatin M (**111**) and excavatolide F (**169**), were isolated. The structure of briavioid A (**166**) was fully confirmed by single-crystal X-ray diffraction analysis [65] (Figure 11).

#### 2.1.6. Aquariane-Type Diterpene

**Figure 12:**
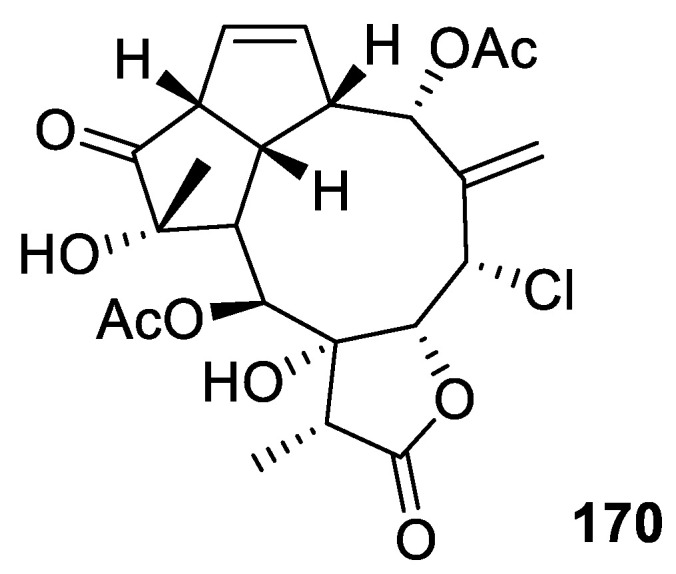
An aquariane compound, aquariolide A (**170**).

Aquariane-type diterpenes possess a tricyclic system that includes a nine-membered ring fused to two five-membered rings [66]. The aquariane scaffold is assumed to be a biosynthetic product of a precursor possessing briarane skeleton by the sequent di-π-methane and vinyl-cyclopropane rearrangements. Aquariolide A (**170**), a new compound isolated from the cultured *Erythropodium caribaeorum*, was an example of an aquariane skeleton [28] (Figure 12).

### 2.2. Biscembranoid

**Figure 13:**
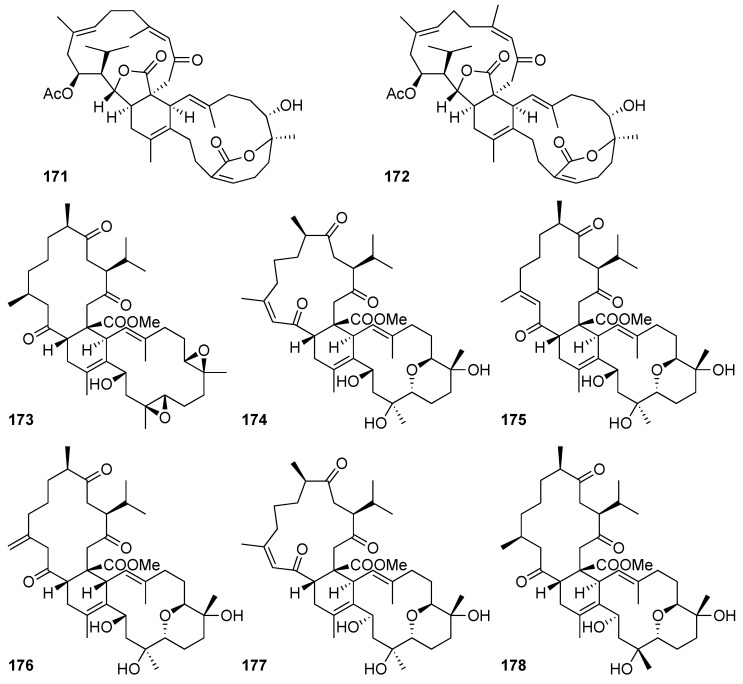
Biscembranoids (**171**–**178**) isolated from the genera *Sarcophyton*.

Biscembranoids are a group of tetraterpenoids that possess a 14-6-14-membered tricyclic system [14]. Commonly, they are biosynthesized by the Diels–Alder reaction of two monocembranoidal units. This type of marine-derived metabolite has been mainly found in the soft corals belonging to the genera *Sarcophyton*, *Lobophytum*, and *Sinularia*. A total of eight biscembranes were isolated from the cultured soft corals of the genera *Sarcophyton* [8,38] (Figure 13).

Two structurally novel biscembranoids, glaucumolides A (**171**) and B (**172**), along with a known biscembrane, ximaolide A (**173**), were isolated from the cultured soft coral *Sarcophyton glaucum*. The structures and absolute configurations of the new metabolites were established using extensive spectroscopic analyses, including a comparison of their circular dichroism spectroscopic data with those of the related compounds. All evidence from the spectroscopic data demonstrated that the two new biscembranes possess an unprecedented molecular scaffold that is biosynthesized by the Diels–Alder reaction between isosarcophytonolide D (**74**) and a novel diene monomer precursor, ε-lactonecembrane [38].

Four new biscembranes, sardigitolides A–D (**174**–**177**), were isolated from the cultured-type *Sarcophyton digitatum*. At the same time, three previously known biscembranoidal derivatives were also purified from the cultivated soft coral and were identified as sarcophytolide L (**178**) and glaucumolides A (**171**) and B (**172**) [8].

### 2.3. Steroid

**Figure 14:**
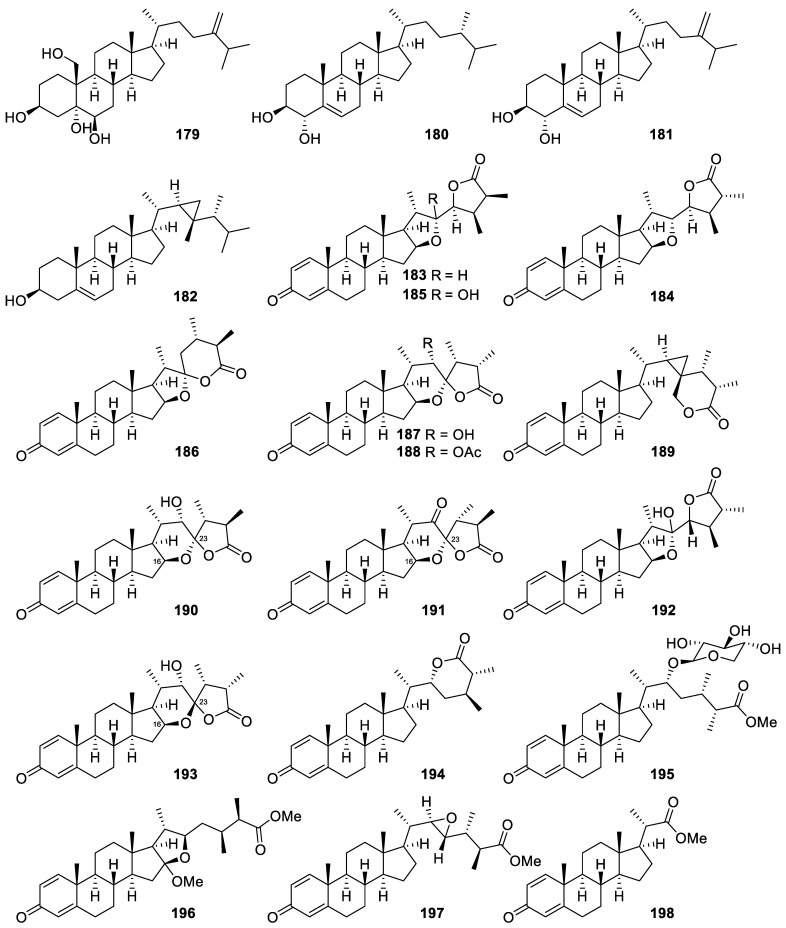
Steroids (**179**–**198**) were isolated from *Litophyton columnaris* and the genera *Sinularia*.

Different types of steroids were obtained from *Litophyton columnaris* and the genera *Sinularia*, including four sterols and sixteen withanolidal and non-withanolidal steroids (Figure 14).

A sterol was isolated from the cultured *Litophyton columnaris* and was identified as nephalsterol A (**179**), which was also previously found in other soft corals of the same genus based on the comparison of its NMR data with those described in the literature [36]. A new sterol, (24S)-24-methylcholest-5-en-3β,4α-diol (**180**), along with two known constituents, 3β,4α-dihydroxyergosta-5,24(28)-diene (**181**) and gorgosterol (**182**), were purified from the cultured-type *Sinularia sandensis*. Single-crystal X-ray diffraction analysis was employed to confirm the absolute configuration of **182** [67].

A chemical investigation demonstrated that the cultured-type soft coral *Sinularia brassica* is an abundant supply of bioactive steroids as compared with the wild type of the same species. A series of sixteen new withanolidal and non-withanolidal steroids, including sinubrasolides A–G (**183**–**189**) [68], sinubrasolides H–L (**190**–**194**) [69], and sinubrasones A–D (**195**–**198**) [70], were purified and identified from the soft coral *Sinularia brassica* cultivated in a tank for five years. The authors also mentioned that sinubrasolides H, I, and K (**190**, **191**, and **193**) shared a common 16,23-oxa-bridged tetrahydropyran framework, which was considered as a novel structure of withanolidal steroid [69]. 

### 2.4. Miscellaneous

In addition to the aforementioned groups, several compounds that contributed to a minor portion of nearly 200 marine metabolites were purified from different species of cultured soft corals, such as a quinone, a hydroquinone, and an α-tocopherol derivative.

**Figure 15:**
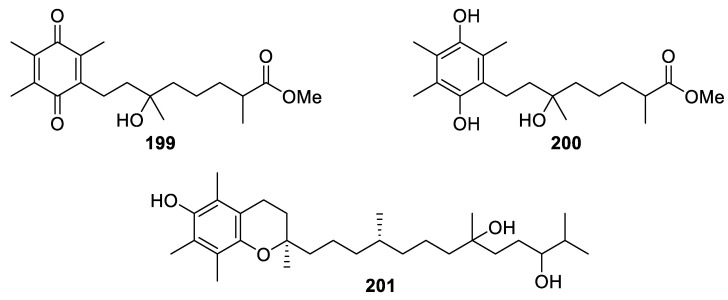
Flexibilisquinone (**199**), sarcotenuhydroquinone (**200**), and crassumtocopherol C (**201**).

A new quinone derivative (Figure 14) was isolated from the cultured soft coral *Sinularia flexibilis* and was designated as flexibilisquinone (**199**) [71]. The organic extract of the cultivated *Sarcophyton tenuispiculatum* yielded a new 1,4-dihydrobenzoquinone, named sarcotenuhydroquinone (**200**) [9]. The ethyl acetate extract of the cultured *Lobophytum crassum* afforded a new α-tocopherol derivative, crassumtocopherol C (**201**) (Figure 15) [7].

## 3. Active Compounds and Their Diverse Bioactivities

### 3.1. Anti-Cancer Activity

The cytotoxicity of a series of new eunicelline-based diterpenes isolated from the cultured soft coral *Klyxum simplex* was evaluated against six human tumor cell lines, including human liver carcinoma (Hep G2 and Hep G3B), human breast carcinoma (MDA-MB-231 and MCF-7), human lung carcinoma (A-549), and human oral cancer cells (Ca9-22). The results showed that only klysimplexins B (**2**) and H (**8**) exhibited a significant cytotoxic effect against a limited panel of cancer cell lines, in which klysimplexin B (**2**) was more potent against Hep G2, Hep 3B, MDA-MB-231, MCF-7, A549, and Ca9-22 cell lines. It was believed that the potent cytotoxic effect of klysimplexin B (**2**) against different tumor cell lines might be caused by the distinguished α,β-unsaturated ketone in its structure when compared with that of the other analogues [24,25,26].

Cembranes are the most popular secondary metabolites that have been found in the chemical investigation of cultured soft corals. Among those, half the isolates displayed anti-cancer effects on various cell lines, contributing to nearly 50% anti-cancer compounds. 11-*epi*-Sinulariolide acetate (**33**) and 11-dehydrosinulariolide (**41**) are the two typical cembranoids that have been subjected to significant anti-cancer research. 11-*epi*-Sinulariolide acetate (**33**), a cembrane isolated from the cultured soft coral *Sinularia flexibilis*, exhibited weak cytotoxicity against the proliferation of MCF-7 cells with ED_50_ of 11.5 μg/mL [29]. However, the compound showed a significant anti-proliferative effect, along with an inhibitory activity on cell migration and invasion, against hepatocellular carcinoma cells (HA22T) in a concentration-dependent manner. The molecular mechanism of anti-metastasis effects of **33** was further clarified by Western blot analysis, which suggested that the cytotoxic effect of this compound was mediated through ERK1/2, p38MAPK, and FAK/PI3K/AKT/mTOR signaling pathways [72]. Another study showed that the anti-proliferative effect of **33** on HA22T cells was apoptosis mediated through mitochondrial dysfunction and endoplasmic reticulum stress-induced pathways [73]. Based on the results, the authors suggested that **33** could be a promising candidate to inhibit metastasis, prevent invasion, and treat hepatocellular carcinoma.

The anti-tumor effect of a cembranoid derivative, 11-dehydrosinulariolide (**41**), on oral cancer cells (CAL-27 and Ca9-22) was thoroughly evaluated by MTT assay, flow cytometry, and wound-healing assay. A comparative proteomic analysis was also performed to compare the expression of overall protein expression in cancer cells treated with 11-dehydrosinulariolide (**41**) and in the control cells. The results showed that 11-dehydrosinulariolide (**41**) reduced the cell viability to 70% and significantly induced both the early and late apoptosis of CAL-27 cells at a concentration of 1.5 µg/mL. Similarly, at a dose of 3.0 μg/mL, the cell viability of Ca9-22 was also reduced to 60%, and the apoptosis of Ca9-22 cells was significantly induced by 11-dehydrosinulariolide (**41**). Additionally, the cell migration of the two cell lines was inhibited in a concentration-dependent manner. The levels of differential proteins related to the apoptosis or inhibition of cancer cell growth were also regulated by 11-dehydrosinulariolide (**41**) in both cell lines [74,75]. In another study, 11-dehydrosinulariolide (**41**) exhibited dose-dependent cytotoxicity against A2058 melanoma cells with the IC_50_ of 5.8 µg/mL. The anti-migratory activity of the compound was investigated with doses ranging from 2 to 6 μg/mL, demonstrating suppression rates of 32%, 51%, and 73% for 2, 4, and 6 μg/mL of 11-dehydrosinulariolide, respectively [76]. The anti-tumor effects of 11-dehydrosinulariolide against human small cell lung cancer cells were also demonstrated in vitro and in vivo experiments [77]. The revealed evidence proved that 11-dehydrosinulariolide is a promising therapeutic agent to be developed as an anti-cancer drug.

Leptoclalin A (**101**), a new spatane-type diterpenoid isolated from the cultured *Sinularia leptoclados*, exhibited cytotoxicity against human tumor cell lines T-47D (IC_50_ = 15.4 μg/mL) and K-562 (IC_50_ = 12.8 μg/mL) [43].

Two norcembranoidal diterpenes, 5-*epi*-sinuleptolide (**102**) and 4α-hydroxy-5-*epi*-sinuleptolide (**104**), were evaluated for their cytotoxicity toward human acute lymphoblastic leukemia (CCRF-CEM). It was concluded that 4α-hydroxy-5-*epi*-sinuleptolide (**104**) was more potent than 5-*epi*-sinuleptolide (**102**) with an IC_50_ value of 4.21 μg/mL due to the presence of a hydroxy substituent at C-4α position [45].

The briarane-type diterpene 2β-acetoxy-2-(debutyryloxy)stecholide E (**140**) showed potent cytotoxicity against P-388 and HT-29 cell lines with ED_50_ values of 0.61 and 6.96 µg/mL, respectively [78].

Three new biscembranoids isolated from the genus *Sarcophyton*, including glaucumolide A (**171**), glaucumolide B (**172**), and sardigitolide B (**171**), showed their cytotoxicity against various cancer cell lines, such as MCF-7, HepG2, MDA-MB-231, HeLa, HL-60, CCRF-CEM, MOLT-4, and K-562 [8,38].

Nephalsterol A (**179**), a known sterol, exhibited cytotoxicity against MOLT-4, SUP-T1, U-937, DLD-1, LNCaP, and MCF7 cells [36].

All six new withanolidal steroids, Sinubrasolides A(**183**), B (**180**), E (**183**), H(**190**), J (**188**), and K (**193**), isolated from *Sinularia brassica* exhibited cytotoxicity against different cell lines (P388, MOLT-4, K-562, and HT-29), evaluated by Alamar Blue assay [68,69].

Four novel non-withanolidal steroids, sinubrasones A–D (**195–198**), were evaluated for their cytotoxicity against a limited panel of cancer cell lines. The authors showed that sinubrasones B and C (**196** and **197**) were more cytotoxic than the other two compounds, and the potent was activity attributed to the presence of a methyl ester at C-25 [70].

Sarcotenuhydroquinone (**200**), a new 1,4-dihydrobenzoquinone, exhibited cytotoxicity toward MCF-7 and MDA-MB-231 cells (IC_50_ = 25.3 ± 2.8 and 36.4 ± 3.6 μM, respectively) [9].

The new α-tocopherol derivative, crassumtocopherol C (**201**), exhibited cytotoxicity against K-562 and Sup-T1 cells (IC_50_ = 34.0 and 23.3 μM, respectively) [7].

### 3.2. Anti-Inflammatory Activity

The bioactivity investigation of klysimplexins I–T (**9**–**20**) showed that klysimplexins J–N, R, and S (**10**–**14**, **18**, and **19**) significantly reduced the expression of iNOS protein at 10 µM. Moreover, klysimplexins R and S (**18** and **19**) also reduced COX-2 expression at the same concentration [7]. Similarly, klysimplexin sulfoxides A–C (compounds **25**–**27**) were also evaluated for their anti-inflammatory activity by the same assays. While klysimplexin sulfoxides A and B (**25** and **26**) significantly inhibited the accumulation of pro-inflammatory iNOS and COX-2 proteins in LPS-stimulated RAW264.7 macrophage cells at 10 µM, the same concentration of klysimplexin sulfoxide C (**27**) showed high inhibitory effect on the expression of both iNOS and COX-2 proteins [27]. The results suggested that those eunicellin-based metabolites could be lead compounds for the development of anti-inflammatory drugs. 

In addition to its cytotoxicity, 11-*epi*-sinulariolide acetate (**33**) was also evaluated for its anti-inflammatory effect. The expression of iNOS protein was reduced to 84.89 ± 8.23%, 39.89 ± 5.64%, 11.8 ± 1.03%, and 1.4 ± 1.74% in the presence of 11-*epi*-sinulariolide acetate (**33**) at 1, 10, 25, and 50 µM, respectively. It also significantly reduced COX-2 levels to 82.89 ± 1.63%, 65.93 ± 4.22%, 52.63 ± 4.76%, and 42.13 ± 3.25% at 10, 25, and 50 µM, respectively [79].

The effect of flexibilide (**34**) on the expression of pro-inflammatory proteins, iNOS and COX-2, and anti-inflammatory transforming growth factor-β (TGF-β) in RAW 264.7 cells induced by lipopolysaccharide was evaluated using Western blot analysis. The results revealed its anti-inflammatory activity through the significant changes in protein levels at 10 μM and 20 μM resulting in the inhibition of iNOS and COX-2, along with the upregulation of TGF-β [80]. 

A series of cembranes isolated from the cultured-type *Sinularia sandensis* and *Sinularia flexibilis* were investigated for their anti-inflammatory activity. The authors suggested that the common seven-membered lactone functional group at position C1 that is shared by the six compounds, including isosinulaflexiolide K (**37**), sinulaflexiolide K (**39**), 11-dehydrosinulariolide (**41**), sinulariolide (**42**), and 3,4:8,11-bisepoxy-7-acetoxycembra-15(17)-en-1,12-olide (**43**), contributed to their inhibitory effect on the expression of both pro-inflammatory proteins, iNOS and COX-2 [32]. 

Briaranes are the diterpene group that possesses the highest number of anti-inflammatory metabolites among the isolates of cultured soft corals. Out of 30 compounds, 21 displayed significantly inhibitory effects on superoxide anion generation and elastase release by human neutrophils, as well as the levels of iNOS and COX-2. The most remarkable bioactive compounds are four new briaranes, including excavatoids I (**131**) and L (**134**), and briaviolides L (**142**) and O (**145**), exhibiting 38.3%, 42.44%, 38.19%, and 89.47% inhibitory effects on elastase release, superoxide anion generation, and the levels of COX-2 and iNOS, respectively [55,56,58,59].

The three novel biscembranoids isolated from *Sarcophyton* spp., glaucumolides A (**171**), B (**172**), and ximaolide A (**173**), exhibited their great inhibitory effect levels of superoxide anion generation and elastase release, as well as the levels of proinflammatory iNOS and COX-2 proteins. The results showed that glaucumolide A (**171**) inhibited superoxide anion generation and elastase release in human neutrophils with the IC_50_ values of 2.79 ± 0.66 and 3.97 ± 0.10 µM, respectively. It also significantly reduced the levels of iNOS and COX-2 to −2.6 ± 2.7 and −0.5 ± 3.2% at 20 μM. Simultaneously, glaucumolide B (**172**) also inhibited superoxide anion generation and elastase release in human neutrophils with the IC_50_ values of 2.79 ± 0.32 and 3.97± 0.10 µM, respectively. The expression of iNOS and COX-2 proinflammatory proteins were reduced to 43.4 ± 5.0 and 6.0 ± 3.6% at 20 μM concentration of glaucumolide B (**172**). Ximaolide A (**173**) reduced the level of COX-2 expression to 22.0 ± 6.5% in LPS-treated macrophage cells at 20 μM [38].

Sinubrasolide A (**183**), a withanolide isolated from the cultured soft coral *Sinularia brassica*, significantly inhibited superoxide anion generation and elastase release in fMLP/CB-stimulated cells with an IC_50_ value of 3.5 ± 0.9 and 1.4 ± 0.1 μM, respectively [69].

Two novel non-withanolidal steroids, sinubrasones C (**197**) and D (**198**), exhibited potent anti-inflammatory effect, which was demonstrated by their ability to suppress superoxide anion generation and elastase release in fMLP/CB-induced human neutrophils. Sinubrasone C (**197**) showed a 58.8% inhibitory effect on superoxide anion generation by fMLP/CB-induced human neutrophils at a concentration of 10 μM while sinubrasone D (**198**) displayed 53.6% and 66.3% inhibitory effect on superoxide anion generation and elastase release, respectively, in fMLP/CB-induced human neutrophils at 10 μM [70].The two sterols isolated from *Sinularia sandensis*, (24S)-24-Methylcholest-5-en-3β,4α-diol (**180**) and gorgosterol (**182**), showed excellent inhibitory effects on the release of iNOS at 10 μM [67].

The new quinone derivative, flexibilisquinone (**199**), significantly suppressed the levels of iNOS and COX-2 at 5–20 µM and 20 µM, respectively [71].

### 3.3. Others

11,12-Epoxy-13,14-dihydroxycembrene-C (**48**) and flaccidoxide (**49**), two cembrane-type metabolites derived from the cultured soft coral *Sinularia gibberosa*, showed significant antimicrobial activity toward *Bacillus subtilis* at the concentrations of 25 and 50 μg/disk, respectively [34].

An in vivo study unveiled that cembranoid 11-*epi*-sinulariolide acetate (**33**) could be a promising agent for the treatment of rheumatic arthritis as it significantly improved the clinical characteristics and the histopathologic features in AIA rat model. The expressions of osteoclast-related proteins, cathepsin K, MMP-9, TRAP, and TNF-a, in the ankle tissues of AIA rats were also reduced in the presence of 11-*epi*-sinulariolide acetate (**33**) [79]. 

The analgesic properties of flexibilide (**34**) were demonstrated in the rat model of carrageenan-induced acute inflammatory pain at a dose of 80 mg/kg [80]. Another study also revealed its anti-neuroinflammatory and analgesic effects in a rat chronic constriction injury model of neuropathic pain [31].

The anti-acne effect of cembranoids derived from the cultured-type *Sinularia flexibilis*, including flexibilide (**34**), 11-dehydrosinulariolide (**41**), and 3,4:8,11-bisepoxy-7-acetoxycembra-15(17)-en-1,12-olide (**43**), was proven by their inhibition against keratinocyte proliferation and inflammatory reactions, which are the main causes of acne. The authors also discussed the structure–activity relationships, in which the heptalactone ring and double bond on carbon 17 were suggested to participate in their anti-acne effect [33].

11-Dehydrosinulariolide (**41**) exhibited a neuroprotective effect, suggesting its potential application for the treatment of Parkinson’s disease [81].

Excavatolide B (**149**), a briarane-type diterpene, not only demonstrated its anti-inflammatory effects in vitro and in vivo but also showed anti-rheumatic effects in adjuvant-induced arthritis (AIA) and type II collagen-induced arthritis (CIA) rat models. That evidence suggested that excavatolide B (**149**) could be a promising candidate for the treatment of rheumatic arthritis in humans [61,62].

Detailed bioactivities of secondary metabolites derived from the cultured soft corals were summarized in Table 1.

## 4. Conclusion and Perspectives

Since 2002 up to the present, a total of 201 compounds, including 170 diterpenes, 8 biscembranoids, 20 steroids, and 3 other miscellaneous compounds have been isolated from different species of cultured-type soft corals belonging to the genera *Sarcophyton*, *Sinularia*, *Briareum*, *Litophyton*, *Lobophytum*, *Klyxum*, and *Erythropodium*. Obviously, diterpenes were the most common compounds found in these marine organisms, representing 84.6% of the isolates. They showed the most diverse structural variations with six subclasses, including eunicellin-based diterpene, cembrane-type diterpene, spatane-type diterpene, norcembranoidal diterpene, briarane-type diterpene, and aquariane-type diterpene. The second highest number of isolates belongs to steroids with 10%, followed by biscembranoids (4%). Most steroids and biscembranoids were discovered as previously unreported compounds (seventeen steroids and six biscembranes). Other quinones and α-tocopherol derivatives were also obtained from the cultured soft corals, which made up nearly 1.5% of the isolated compounds. Generally, novel compounds occupied nearly 70% of the isolates, showing anti-tumor and anti-inflammatory effects. Many isolated compounds displayed weak or moderate cytotoxicity, but it should be noted that only a limited panel of cancer cell lines was utilized to evaluate the cytotoxicity of the secondary metabolites, while there are hundreds of cell lines that were not used for the screening of the cytotoxic properties. Other biological properties were also observed in the isolated derivatives, including anti-acne, anti-rheumatic, antinociceptive, antibacterial activities, and neuroprotective effects. 

According to our literature-based investigation, only pseudopterosins derived from the soft coral *Pseudopterogorgia elisabethae* were subjected to clinical trials for their anti-inflammatory and analgesic effects and were successfully commercialized as a cosmetic product for human. It is remarkable that the supply of pseudopterosins is still derived from the exploitation of the wild-type soft coral in the Bahamas, which may lead to an upcoming supply problem [84]. Until now, no metabolite derived from aquaculture soft corals has been approved for clinical use as a therapeutic agent despite their pharmacological potential because the large-scale production of marine natural products for clinical trials has not been resolved effectively. The supply issue can be handled by the chemical synthesis of the potential compounds or aquaculture of the soft corals. Due to the structure complexity and unknown biosynthetic pathways of marine secondary metabolites, chemical synthesis may be more difficult and time- and resource-consuming than aquaculture methods. Consequently, coral aquaculture has attracted great interests regarding the discovery of advanced technologies for the optimization of soft coral mass production. So far, mariculture (”in situ” aquaculture—sea based) and captive breeding (“ex situ” aquaculture—aquarium based) have been the two main methods of coral aquaculture [6]. Moreover, biotechnological techniques have also been available to support coral aquaculture, including genetic manipulation and probiotics use. These advances can increase nutrition, mitigate coral diseases, and enhance the gene expression for the biosynthesis of the target metabolites to improve the production of potential compounds [85,86].

The anti-inflammatory effects of 11-epi-Sinulariolide acetate (**33**) were investigated in both in vitro and in vivo tests, which suggested that it is one of the tremendous candidates deserving further development as a therapeutic agent of rheumatic diseases and other inflammatory diseases [79]. In another investigation, a fraction derived from the ethyl acetate extract of the cultured *Sinularia flexibilis* displayed its anti-acne properties on an in vivo model—*Cutibacterium acnes*-induced Wistar rats. Concomitantly, the isolates derived from this fraction, including flexibilide (sinularin) (**34**), 11-Dehydrosinulariolide (**41**), and 3,4:8,11-Bisepoxy-7-acetoxycembra-15(17)-en-1,12-olide (**43**) also exhibited their anti-keratinocyte proliferation and anti-inflammatory actions in various in vitro studies [33]. All these results support the development of marine natural products derived from the extract of aquaculture *Sinularia flexibilis* or the three bioactive secondary metabolites.

The productivity of the cultured types could be even much higher than those of the wild types. As a typical example of high-yield production of natural products from cultured soft corals, the cultured *Briareum stechei* afforded about 0.76 g/kg of excavatolide B, which was 3-fold higher than the yield of about 0.24 g/kg from the wild-type [61]. Aquaculture not only provides a sustainable resource for soft corals to investigate their secondary metabolites but may also stimulate the species to produce different compounds in comparison with the wild type corals. For instance, the cultured-type *Klyxum simplex* yielded klysimplexins A–H, while none of these compounds were found in the wild-type soft coral and simplexins A–I were found instead [24]. Another example is from the case of *Sinularia flexibilis*.The wild type produced flexibilisolide B and flexibilisin B while the cultivated soft coral yielded other two metabolites, flexibilisolide A (**31**) and flexibilisin A (**32**) [29]. These differences suggest that farming soft corals could increase the structure diversity of natural products and the possibility of discovering new bioactive substances as well. 

## Figures and Tables

**Table 1:** Bioactive compounds isolated from the culture-type soft corals.

Compound	Chemical Classification	Novelty	Source	Bioactivities
Klysimplexin B (**2**)	Eunicellin-based diterpene	New	*Klyxum simplex*	Exhibited moderate cytotoxicity against Hep G2, Hep 3B, MDA-MB-231, MCF-7, A549, and Ca9-22 cell lines (IC_50_ = 3.0, 3.6, 6.9, 3.0, 2.0, and 1.8 μg/mL, respectively) [24]
Klysimplexin H (**8**)	Eunicellin-based diterpene	New	*Klyxum simplex*	Showed cytotoxicity against Hep G2, Hep 3B, MDA-MB-231, MCF-7, A549, and Ca9-22 cell lines (IC_50_ = 5.6, 6.9, 4.4, 5.6, 2.8 and 6.1 μg/mL, respectively) [24]
Klysimplexin J (**10**)	Eunicellin-based diterpene	New	*Klyxum simplex*	Significantly reduced the expression of iNOS protein at 10 μM [25]
Klysimplexin K (**11**)	Eunicellin-based diterpene	New	*Klyxum simplex*	Significantly reduced the expression of iNOS protein at 10 μM [25]
Klysimplexin L (**12**)	Eunicellin-based diterpene	New	*Klyxum simplex*	Significantly reduced the expression of iNOS protein at 10 μM [25]
Klysimplexin M (**13**)	Eunicellin-based diterpene	New	*Klyxum simplex*	Significantly reduced the expression of iNOS protein at 10 μM [25]
Klysimplexin N (**14**)	Eunicellin-based diterpene	New	*Klyxum simplex*	Significantly reduced the expression of iNOS protein at 10 μM [25]
Klysimplexin Q (**17**)	Eunicellin-based diterpene	New	*Klyxum simplex*	Exhibited cytotoxicity against Hep G2, Hep 3B, MDA-MB-231, MCF-7, A549, and Ca9-22 cell lines (IC_50_ = 53.2, 35.1, 44.0, 36.5, 40.5, and 40.5 μM, respectively) [25]
Klysimplexin R (**18**)	Eunicellin-based diterpene	New	*Klyxum simplex*	Significantly reduced the expression of iNOS and COX-2 proteins at 10 μM [25]
Klysimplexin S (**19**)	Eunicellin-based diterpene	New	*Klyxum simplex*	Significantly reduced the expression of iNOS and COX-2 proteins at 10 μM [25]
Klysimplexin T (**20**)	Eunicellin-based diterpene	New	*Klyxum simplex*	Showed cytotoxicity against the growth of Hep G2, Hep 3B, MDA-MB-231, MCF-7, A549, and Ca9-22 cells (IC_50_ = 34.3, 26.4, 44.0, 27.2, 42.0 and 37.4 μM, respectively) [25]
Klysimplexin sulfoxide A (**25**)	Eunicellin-based diterpene	New	*Klyxum simplex*	Significantly reduced the expression of iNOS protein at 10 μM [27]
Klysimplexin sulfoxide B (**26**)	Eunicellin-based diterpene	New	*Klyxum simplex*	Significantly reduced the expression of iNOS protein at 10 μM [27]
Klysimplexin sulfoxide C (**27**)	Eunicellin-based diterpene	New	*Klyxum simplex*	Significantly reduced the expression of iNOS and COX-2 proteins at 10 μM [27]
11-*epi*-Sinulariolide acetate (**33**)	Cembrane-type diterpene	Known	*Sinularia flexibilis*	Exhibited weak cytotoxicity against the proliferation of MCF-7 cells (ED_50_ = 11.5 μg/mL) [29] Showed a significant antiproliferative effect and inhibitory activity on cell migration and invasion in HA22T cells in a concentration-dependent manner [72] Induced apoptosis in HA22T cells [73] Significantly reduced iNOS levels to 84.89 ± 8.23%, 39.89 ± 5.64%, 11.8 ± 1.03%, and 1.4 ± 1.74% at concentrations of 1, 10, 25, and 50 μM, respectively [79] Significantly reduced COX-2 levels to 82.89 ± 1.63%, 65.93 ± 4.22%, 52.63 ± 4.76%, and 42.13 ± 3.25% at 10, 25, and 50 μM, respectively [79] Anti-rheumatic effect: Significantly reduced the clinical characteristics and the expressions of osteoclast-related proteins and improved the histopathologic features in the AIA rat model [79]
Flexibilide (sinularin) (**34**)	Cembrane-type diterpene	Known	*Sinularia flexibilis*	Exhibited significant cytotoxicity against KB and PS cell lines (ED50 = 0.3 μg/mL) [82] Significantly reduced iNOS levels to 53.45 ± 3.27%, 36.45 ± 5.15%, 33.38 ± 4.61%, and 19.48 ± 3.95% at 0.1, 1, 10, and 20 μM, respectively [80] Significantly reduced the levels of COX-2 protein to 82.72 ± 6.17% and 66.23 ± 3.27% at 0.1, 1, 10, and 20 μM, respectively [80] Significantly increased the levels of TGF-β protein to 137.75 ± 5.97%, 149.82 ± 6.15%, 142.71 ± 4.57%, and 138.02 ± 5.15% at 0.1, 1, 10, and 20 μM, respectively [80] Exhibited analgesic properties in a rat model at 80 mg/kg [80] Exerted anti-neuroinflammatory and analgesic effects in a rat model [31] Anti-acne capabilities [33]
Isosinulaflexiolide K (**37**)	Cembrane-type diterpene	New	*Sinularia flexibilis*	Significantly reduced the levels of iNOS and COX-2 proteins to 30.9 ± 4.1% and 47.1 ± 3.8%, respectively, at 10 μM [32]
Sinulaflexiolide K (**39**)	Cembrane-type diterpene	Known	*Sinularia flexibilis*	Significantly reduced the levels of iNOS and COX-2 proteins to 37.4 ± 5.9% and 51.4 ± 5.6%, respectively, at 10 μM [32]
(−)-Sandensolide (**40**)	Cembrane-type diterpene	Known	*Sinularia flexibilis*	Significantly reduced the levels of iNOS and COX-2 proteins to 61 ± 3.4% and 51.9 ± 7.2%, respectively, at 10 μM [32]
11-Dehydrosinulariolide (**41**)	Cembrane-type diterpene	Known	*Sinularia flexibilis*	Reduced the cell viability to 70% and significantly induced both the early and late apoptosis of CAL-27 cells at a concentration of 1.5 µg/mL [74] Reduced the cell viability to 60% and significantly induced both the early and late apoptosis of Ca9-22 cells at a concentration of 3.0 µg/mL [75] Inhibited cell migration of CAL-27 and Ca9-22 cells in a dose-dependent manner [74,75] Exhibited a dose-dependent (2–8 μg/mL) cytotoxicity against A2058 cells (IC_50_ = 5.8 μg/mL) [76] Exhibited a dose-dependent anti-migratory effect against A2058 cells with the suppression rates of approximately 32%, 51% and 73% for 2, 4, and 6 μg/mL, respectively [76] Exhibited cytotoxic against H1688 and H146 cell (after 12, 24, and 48 h of exposure, IC_50_ = >50, 29.8 ± 3.4, and 19.1 ± 2.4 µM respectively, for H1688 cells, and > 50, 43.5 ± 6.6, and 25.1 ± 2.6 µM, respectively, for H146 cells) [77] Significantly suppressed the H1688 tumor growth in a mouse xenograft model with an intraperitoneal injection regimen of 10 mg/kg x 3 times/week for 22 days [77] Significantly reduced the levels of iNOS and COX-2 proteins to 31.9 ± 5.1% and 49 ± 5.6%, respectively, at 10 μM [32] Anti-acne capabilities [33] Neuroprotective effect [81]
Sinulariolide (**42**)	Cembrane-type diterpene	Known	*Sinularia flexibilis*	Exhibited weak cytotoxicity in MCF-7 cells (ED_50_ = 16.9 μg/mL) [29] Exhibited cytotoxicity against KB and PS cell lines (ED50 = 20 and 7 μg/mL, respectively) [82] Inhibited cell proliferation, suppressed A375 melanoma cell migration, and elevated early and late apoptosis in a concentration-dependent manner [83] Significantly reduced the levels of iNOS and COX-2 proteins to 47.7 ± 6.3% and 52.2 ± 5.1%, respectively, at 10 μM [32]
3,4:8,11-Bisepoxy-7-acetoxycembra-15(17)-en-1,12-olide (**43**)	Cembrane-type diterpene	Known	*Sinularia flexibilis*	Significantly reduced the levels of iNOS and COX-2 proteins to 25.7 ± 5.2% and 55.3 ± 8.2%, respectively, at 10 μM [32] Anti-acne capabilities [33]
Dihydrosinularin (**46**)	Cembrane-type diterpene	Known	*Sinularia flexibilis*	Exhibited cytotoxicity against KB and PS cell lines (ED50 = 16 and 1.1 μg/mL, respectively) [82]
11,12-Epoxy-13,14-dihydroxycembrene-C (**48**)	Cembrane-type diterpene	Known	*Sinularia gibberosa*	Exhibited significant antibacterial activity at a concentration of 25 μg/disk [34]
Flaccidoxide (**49**)	Cembrane-type diterpene	Known	*Sinularia gibberosa*	Exhibited significant antibacterial activity at a concentration of 50 μg/disk [34]
Columnariol A (**56**)	Cembrane-type diterpene	New	*Litophyton columnaris*	Exhibited moderate cytotoxicity toward LNCaP cells (IC_50_ = 9.80 μg/mL) [35] Significantly reduced the levels of iNOS and COX-2 at 50 μM [35]
Columnariol B (**57**)	Cembrane-type diterpene	New	*Litophyton columnaris*	Significantly reduced the levels of iNOS and COX-2 at 50 μM [35]
Culobophylin A (**61**)	Cembrane-type diterpenoid	New	*Lobophytum crassum*	Exhibited cytotoxicity against the HL60, MDA-MB-231, DLD-1, and HCT-116 cells (IC_50_ = 3.0, 16.8, 4.6, and 16.3 μg/mL, respectively) [37]
Culobophylin B (**62**)	Cembrane-type diterpenoid	New	*Lobophytum crassum*	Exhibited cytotoxicity against HL60, DLD-1 and HCT-116 cells (IC_50_ = 6.8, 16.2, and 16.7 μg/mL, respectively) [37]
Lobophylin B (**67**)	Cembrane-type diterpenoid	Known	*Lobophytum crassum*	Exhibited cytotoxicity against K562, Molt 4, and Sup-T1 cells (IC_50_ = 16.3, 12.3, and 4.6 μM, respectively) [7]
Lobocrassin B (**68**)	Cembrane-type diterpenoid	Known	*Lobophytum crassum*	Exhibited cytotoxicity against K562, Molt 4, U937, and Sup-T1 cells (IC_50_ =3.3, 2.3, 5.2, and 6.2 μM, respectively) [7]
Lobocrassin C (**69**)	Cembrane-type diterpenoid	Known	*Lobophytum crassum*	Exhibited cytotoxicity toward Sup-T1 cells (IC_50_ = 35.8 μM) [7]
Crassocolide E (**70**)	Cembrane-type diterpenoid	Known	*Lobophytum crassum*	Exhibited cytotoxicity against K562, Molt 4, U937, and Sup-T1 cells (IC_50_ = 11.3, 6.2, 15.8, and 5.2 μM, respectively) [7]
Sarcocrassolide (**71**)	Cembrane-type diterpenoid	Known	*Lobophytum crassum*	Exhibited cytotoxicity against K562, Molt 4, U937, and Sup-T1 cells (IC_50_ = 18.1, 8.4, 4.4, and 8.3 μM, respectively) [7]
13-Acetoxysarcocrassolide (**72**)	Cembrane-type diterpenoid	Known	*Lobophytum crassum*	Exhibited cytotoxicity against K562, Molt 4, U937, and Sup-T1 cells (IC_50_ = 3.3, 1.2, 7.1, and 1.5 μM, respectively) [7]
Sarcocrassocolide F (**73**)	Cembrane-type diterpenoid	Known	*Lobophytum crassum*	Exhibited cytotoxicity toward K562, Molt 4, U937, and Sup-T1 cells (IC_50_ =12.3, 4.8, 10.9, and 6.1 μM, respectively) [7]
Sarcocrassocolide G (**74**)	Cembrane-type diterpenoid	Known	*Lobophytum crassum*	Exhibited cytotoxicity against K562, Molt 4, U937, and Sup-T1 cells (IC_50_ =13.0, 7.0, 23.3, and 6.6 μM, respectively) [7]
Sarcocrassocolide M (**75**)	Cembrane-type diterpenoid	Known	*Lobophytum crassum*	Exhibited cytotoxicity against K562, Molt 4, U937, and Sup-T1 cells (IC_50_ =15.3, 11.6, 32.0, and 10.2 μM, respectively) [7]
14-Deoxycrassin (**76**)	Cembrane-type diterpenoid	Known	*Lobophytum crassum*	Exhibited cytotoxicity against K562, Molt 4, U937, and Sup-T1 cells (IC_50_ =4.5, 2.9, 7.0, and 4.5 μM, respectively) [7]
Isosarcophytonolide D (**77**)	Cembrane-type diterpenoid	Known	*Sarcophyton digitatum* *Sarcophyton glaucum*	Exhibited cytotoxicity against MCF-7 carcinoma cell lines (IC_50_ = 10.9 ± 4.3 μg/mL) [8] Inhibited LPS-induced IL-1β production to 56 ± 1% at 10 µg/mL (IC_50_ = 14.9 ± 5.1 µg/mL) [8] Exhibited cytotoxicity against HL-60, CCRF-CEM, and MOLT-4 cell lines (ED_50_ = 13.0 ± 1.9, 15.3 ± 2.5, and 17.2 ± 3.1 μg/mL, respectively) [38]
Sarcotenusene A (**79**)	Cembrane-type diterpenoid	New	*Sarcophyton tenuispiculatum*	Exhibited cytotoxicity against MCF-7 cells (IC_50_ = 34.3 ± 3.7 μM) [9]
Sarcophytonin F (**83**)	Cembrane-type diterpenoid	Known	*Sarcophyton tenuispiculatum*	Exhibited cytotoxicity against MCF-7 and MDA-MB-231 cells (IC_50_ = 30.1 ± 3.1 and 38.6 ± 5.0 μM, respectively) [9]
(2S,7S,8S)-Sarcophytoxide (**84**)	Cembrane-type diterpenoid	Known	*Sarcophyton tenuispiculatum*	Exhibited cytotoxicity against MCF-7 and HepG2 cells (IC_50_ = 37.6 ± 4.2 and 35.2 ± 4.4 μM, respectively) [9]
(2S,7R,8R)-Sarcophytoxide (**85**)	Cembrane-type diterpenoid	Known	*Sarcophyton tenuispiculatum*	Exhibited cytotoxicity against MCF-7 and HepG2 cells (IC_50_ = 33.3 ± 3.5 and 28.6 ± 3.4 μM, respectively) [9]
3,4-Dihydro-4α-hydroxy-∆2-sarcophine (**87**)	Cembrane-type diterpenoid	Known	*Sarcophyton tenuispiculatum*	Exhibited cytotoxicity against MCF-7 and HepG2 cells (IC_50_ = 24.3 ± 3.0 and 34.5 ± 4.2 μM, respectively) [9]
(+)-7α,8β-Dihydroxydeepoxysarcophine (**89**)	Cembrane-type diterpenoid	Known	*Sarcophyton tenuispiculatum*	Inhibited IL-1β production to 56 ± 1% in LPS-stimulated murine macrophage J774A.1 cell at 30 µM [9]
A hydroperoxide obtained by autoxidation of dihydrofuranocembranoid (**91**)	Cembrane-type diterpenoid	Known	*Sarcophyton tenuispiculatum*	Exhibited cytotoxicity against MCF-7 and HepG2 cells (IC_50_ = 27.2 ± 4.0 and 36.4 ± 5.3 μM, respectively) [9]
Briaviotriol A (**94**)	Cembrane-type diterpenoid	New	*Briareum violaceum*	Reduced the levels of iNOS to 67.7 ± 2.4% at 10 μM [40]
Briaviotriol B (**95**)	Cembrane-type diterpenoid	New	*Briareum violaceum*	Reduced the levels of iNOS to 79.5 ± 9.4% at 10 μM [40]
Briaviodiol A (**96**)	Cembrane-type diterpenoid	Known	*Briareum violaceum*	Reduced the levels of iNOS to 61.9 ± 7.3% at 10 μM [40]
Briaviodiol B (**97**)	Cembrane-type diterpenoid	New	*Briareum violaceum*	Reduced the level of iNOS to 43 ± 6% at 10 μM [41]
Briaviodiol D (**99**)	Cembrane-type diterpenoid	New	*Briareum violaceum*	Reduced the level of iNOS to 61 ± 7% at 10 μM [41]
Briaviodiol E (**100**)	Cembrane-type diterpenoid	New	*Briareum violaceum*	Reduced the level of iNOS to 46 ± 10% at 10 μM [41]
Leptoclalin A (**101**)	Spatane-type diterpenoid	New	*Sinularia leptoclados*	Exhibited weak cytotoxicity against human tumor cell lines T-47D (IC_50_ = 15.4 μg/mL) and K-562 (IC_50_ = 12.8 μg/mL) [43]
5-*epi*-Sinuleptolide (**102**)	Norcembranoidal diterpene	Known	*Sinularia numerosa*	Exhibited cytotoxicity against CCRF-CEM and HL-60 cells (IC_50_ = 11.07 and 11.11 μg/mL, respectively) [45]
4α-Hydroxy-5-*epi*-sinuleptolide (**104**)	Norcembranoidal diterpene	New	*Sinularia numerosa*	Exhibited cytotoxicity against CCRF-CEM, HL-60, K-562, and U-937 cells (IC_50_ = 4.21, 10.38, 18.07, and 10.08 μg/mL, respectively) [45]
Excavatolide C (**109**)	Briarane-type diterpene	Known	*Briareum stechei*	Showed a 15.47% inhibitory effect on superoxide anion generation by human neutrophils at 10 μg/mL [48]
Briaexcavatin P (**114**)	Briarane-type diterpene	New	*Briareum stechei*	Showed a 14.99% inhibitory effect on superoxide anion generation by human neutrophils at 10 μg/mL [49]
Briaexcavatin S (**117**)	Briarane-type diterpene	New	*Briareum stechei*	Exhibited weak cytotoxicity toward CCRF-CEM cells (ED_50_ = 37.8 μg/mL) [50]
Briaexcavatin V (**120**)	Briarane-type diterpene	New	*Briareum stechei*	Showed 11.39 ± 1.26% and 23.27 ± 8.65% inhibitory effects on superoxide anion generation and elastase release by human neutrophils at 10 μg/mL, respectively [52]
Briaexcavatin X (**122**)	Briarane-type diterpene	New	*Briareum stechei*	Displayed a 13.69 ± 3.84% inhibitory effect on superoxide anion generation by human neutrophils at 10 μg/mL [52]
Briaexcavatin Y (**123**)	Briarane-type diterpene	New	*Briareum stechei*	Displayed a 17.47 ± 0.85% inhibitory effect on superoxide anion generation by human neutrophils at 10 μg/mL [52]
Excavatoid E (**127**)	Briarane-type diterpene	New	*Briareum stechei*	Exhibited 26.22 ± 0.50% and 12.95 ± 6.99% inhibitory effects on elastase release and superoxide anion generation by human neutrophils at 10 μg/mL, respectively [54]
Excavatoid F (**128**)	Briarane-type diterpene	New	*Briareum stechei*	Exhibited a 30.63 ± 4.68% inhibitory effect on elastase release by human neutrophils at 10 μg/mL [54]
Excavatoid H (**130**)	Briarane-type diterpene	New	*Briareum stechei*	Exhibited cytotoxicity against CCRF-CEM cells (ED_50_ = 13.1 μg/mL) [55]
Excavatoid I (**131**)	Briarane-type diterpene	New	*Briareum stechei*	Displayed 38.3% and 21.8% inhibitory effects on elastase release and superoxide anion generation by human neutrophils at 10 μg/mL, respectively [55]
Excavatoid L (**134**)	Briarane-type diterpene	New	*Briareum stechei*	Displayed 42.44 ± 2.38% and 31.25 ± 0.07% inhibitory effects on superoxide anion generation and elastase release by human neutrophils at 10 μg/mL, respectively [56]
Excavatoid M (**135**)	Briarane-type diterpene	New	*Briareum stechei*	Displayed 14.85 ± 3.66% and 16.96 ± 2.93% inhibitory effects on superoxide anion generation and elastase release by human neutrophils at 10 μg/mL, respectively [56]
Excavatoid N (**136**)	Briarane-type diterpene	New	*Briareum stechei*	Displayed 10.90 ± 0.50% and 22.21 ± 3.34% inhibitory effects on superoxide anion generation and elastase release by human neutrophils at 10 μg/mL, respectively [56]
Excavatoid O (**137**)	Briarane-type diterpene	New	*Briareum stechei*	Displayed a 16.9% inhibitory effect on elastase release by human neutrophils at 10 μg/mL [57]
Excavatoid P (**138**)	Briarane-type diterpene	New	*Briareum stechei*	Displayed a 16.1% inhibitory effect on elastase release by human neutrophils at 10 μg/mL [57]
Briarenolide D (**139**)	Briarane-type diterpene	New	*Briareum stechei*	Showed moderate cytotoxicity toward DLD-1 and CCRF-CEM cells (ED_50_ = 9.6 and 6.9 μg/mL, respectively) [47]
2β-Acetoxy-2-(debutyryloxy)stecholide E (**140**)	Briarane-type diterpene	Known	*Briareum stechei*	Showed cytotoxicity against P-388 and HT-29 cell lines (ED_50_ = 0.61 and 6.96 μg/mL, respectively) [78]
Briaviolide L (**142**)	Briarane-type diterpene	New	*Briareum violaceum*	Reduced the levels of iNOS and COX-2 to 46.68% and 61.81%, respectively, at 33.7 μM [58]
Briaviolide O (**145**)	Briarane-type diterpene	New	*Briareum violaceum*	Reduced the levels of iNOS and COX-2 to 10.53 ± 1.38% and 84.31 ± 2.14%, respectively, at 10 μM [59]
Briaviolide P (**146**)	Briarane-type diterpene	New	*Briareum violaceum*	Reduced the level of COX-2 to 87.83 ± 3.36% at 10 μM [59]
Briaviolide Q (**147**)	Briarane-type diterpene	New	*Briareum violaceum*	Reduced the level of iNOS to 26.4 ± 1.5% at 10 μM [60]
Excavatolide Z (**148**)	Briarane-type diterpene	Known	*Briareum violaceum*	Reduced the level of iNOS to 66.2 ± 9.6% at 10 μM [60]
Excavatolide B (**149**)	Briarane-type diterpene	Known	*Briareum stechei*	Showed significant dose-dependent inhibition of iNOS gene expression in RAW 264.7 murine macrophages at doses of 1, 10, 25, and 50 μM [19] Significantly inhibited COX-2 gene expression in RAW 264.7 murine macrophages at 25 and 50 μM [61] Reduced the infiltration of inflammatory cells and iNOS protein expression to ameliorate the pain behavior and inflammatory response in carrageenan-induced inflammatory rats at 15 or 60 mg/kg [61] Anti-rheumatic effect: Reduced osteoclastogenesis via the downregulation of the inflammatory factors IL-17A and M-CSF to influence the MAPK and HO-1/HMGB-1 pathways in AIA and CIA rats [62]
Briarenol P (**151**)	Briarane-type diterpene	New	*Briareum stechei*	Reduced the expressions of iNOS and β-actin to 88.24 ± 7.51% and 85.40 ± 5.35% at 10 μM [46]
Briarenol S (**154**)	Briarane-type diterpene	New	*Briareum stechei*	Inhibited the release of iNOS to 78.50% [63]
Briarenol T (**155**)	Briarane-type diterpene	New	*Briareum stechei*	Inhibited the release of iNOS to 79.95% [63]
Briavioid A (**166**)	Briarane-type diterpene	New	*Briareum violaceum*	Reduced the release of iNOS protein to 77.50% at 10 μM [65]
Briavioid B (**167**)	Briarane-type diterpene	New	*Briareum violaceum*	Reduced the release of iNOS protein to 80.24% at 10 μM [65]
Briavioid C (**168**)	Briarane-type diterpene	New	*Briareum violaceum*	Reduced the release of iNOS protein to 71.30% at 10 μM [65]
Excavatolide F (**169**)	Briarane-type diterpene	Known	*Briareum violaceum*	Significantly reduced the release of iNOS protein to 28.60% at 10 μM [65]
Glaucumolide A (**171**)	Biscembranoid	New	*Sarcophyton digitatum* *Sarcophyton glaucum*	Exhibited cytotoxicity against MCF-7, HepG2, and HeLa carcinoma cell lines (IC_50_ = 10.1 ± 3.3, 14.9 ± 3.5, and 17.1 ± 4.5 μg/mL, respectively) [8] Inhibited LPS-induced IL-1β production to 68 ± 1% at 10 µg/mL (IC_50_ = 10.7 ± 2.7 µg/mL) [8] Exhibited cytotoxicity against HL-60, CCRF-CEM, MOLT-4, and K-562 cell lines (ED_50_ = 6.6 ± 1.2, 7.4 ± 1.5, 11.0 ± 2.8, and 19.2 ± 2.3 μg/mL, respectively) [38] Inhibited superoxide anion generation and elastase release in human neutrophils (IC_50_ = 2.79 ± 0.66 and 3.97± 0.10 µM, respectively) [38] Significantly reduced the levels of iNOS and COX-2 to 59.4 ± 9.0 and 66.5 ± 4.4%; 31.3 ± 6.5 and 78.3 ± 5.0%; and −2.6 ± 2.7 and −0.5 ± 3.2% at concentrations of 5, 10, and 20 μM, respectively [38]
Glaucumolide B (**172**)	Biscembranoid	New	*Sarcophyton digitatum* *Sarcophyton glaucum*	Exhibited cytotoxicity against MCF-7, MDA-MB-231, and HepG2 carcinoma cell lines (IC_50_ = 9.4 ± 3.0, 17.8 ± 4.5, and 14.9 ± 4.2 μg/mL, respectively) [8] Exhibited cytotoxicity against HL-60, CCRF-CEM, MOLT-4, and K-562 cell lines (ED_50_ = 3.8 ± 0.9, 5.3 ± 1.4, 11.0 ± 2.2, and 12.6 ± 0.7 μg/mL, respectively) [38] Inhibited superoxide anion generation and elastase release in human neutrophils (IC_50_ = 2.79 ± 0.32 and 3.97± 0.10 µM, respectively) [38] Significantly reduced the levels of iNOS and COX-2 to 75.9 ± 3.5 and 64.3 ± 6.9%; and 43.4 ± 5.0 and 6.0 ± 3.6% at concentrations of 10 and 20 μM, respectively [38]
Ximaolide A (**173**)	Biscembranoid	Known	*Sarcophyton glaucum*	Reduced the level of COX-2 expression to 22.0 ± 6.5% in LPS-treated macrophage cells at 20 μM [38]
Sardigitolide B (**175**)	Biscembranoid	New	*Sarcophyton digitatum*	Exhibited cytotoxicity against MCF-7 and MDA-MB-231 carcinoma cell lines (IC_50_ = 9.6 ± 3.0 and 14.8 ± 4.0 μg/mL, respectively) [8]
Nephalsterol A (**179**)	Sterol	Known	*Litophyton columnaris*	Exhibited cytotoxicity against MOLT-4, SUP-T1, U-937, DLD-1, LNCaP, and MCF7 cells (IC_50_ = 22.5, 32.4, 38.6, 44.2, 11.6, and 9.8 μM, respectively) [36]
(24S)-24-Methylcholest-5-en-3β,4α-diol (**180**)	Sterol	New	*Sinularia sandensis*	Reduced the release of iNOS to 89.52 ± 3.37% at 10 μM [67]
Gorgosterol (**182**)	Sterol	Known	*Sinularia sandensis*	Reduced the release of iNOS to 87.34 ± 2.48% at 10 μM [67]
Sinubrasolide A (**183**)	Withanolidal steroid	New	*Sinularia brassica*	Exhibited cytotoxicity against P388, MOLT-4, K-562, and HT-29 cells (IC_50_ = 29.9 ± 3.0, 12.1 ± 1.1, 8.7 ± 1.4, and 18.7 ± 2.5 μM, respectively) [69] Showed potent inhibitory effect against superoxide anion generation and elastase release in fMLP/CB-stimulated cells (IC_50_ = 3.5 ± 0.9 and 1.4 ± 0.1 μM, respectively) [69]
Sinubrasolide B (**184**)	Withanolidal steroid	New	*Sinularia brassica*	Exhibited cytotoxicity against P388, MOLT 4, and HT-29 cancer cell lines (ED_50_ = 9.1 ± 1.4, 4.8 ± 0.9, and 4.8 ± 0.7 μM, respectively) [68]
Sinubrasolide E (**187**)	Withanolidal steroid	New	*Sinularia brassica*	Showed cytotoxicity against MOLT 4 and HT-29 cell lines (ED_50_ = of 9.9 ± 1.8 and 7.5 ± 1.5 μM, respectively) [68]
Sinubrasolide H (**190**)	Withanolidal steroid	New	*Sinularia brassica*	Exhibited cytotoxicity against P388, MOLT-4, K-562, and HT-29 cells (IC_50_ = 39.8 ± 7.7, 28.6 ± 5.9, 29.7 ± 8.6, and 24.4 ± 6.2 μM, respectively) [69]
Sinubrasolide J (**192**)	Withanolidal steroid	New	*Sinularia brassica*	Exhibited cytotoxicity against P388, MOLT-4, K-562, and HT-29 cells (IC_50_ = 18.7 ± 3.1, 17.2 ± 1.5, 12.6 ± 3.1, and 11.2 ± 1.1 μM, respectively) [69]
Sinubrasolide K (**193**)	Withanolidal steroid	New	*Sinularia brassica*	Exhibited cytotoxicity against P388, MOLT-4, K-562, and HT-29 cells (IC_50_ = 18.3 ± 2.6, 13.7 ± 3.3, 17.4 ± 3.3, and 20.5 ± 3.7 μM, respectively) [69]
Sinubrasone B (**196**)	Non-withanolidal Steroid	New	*Sinularia brassica*	Showed significant cytotoxicity against P388D1, MOLT-4, K-562, and HT-29 cells (IC_50_ = 9.7 ± 1.2, 6.0 ± 0.4, 5.2 ± 0.8, and 7.6 ± 2.3 μM, respectively) [70]
Sinubrasone C (**197**)	Non-withanolidal Steroid	New	*Sinularia brassica*	Showed significant cytotoxicity against P388D1, MOLT-4, K-562, and HT-29 cells (IC_50_ = 5.7 ± 1.8, 5.3 ± 1.3, 12.1 ± 2.4, and 10.4 ± 2.2 μM, respectively) [70] Inhibited elastase release in fMLP/CB-induced human neutrophils (IC_50_ = 6.6 ± 1.1 μM) [70]
Sinubrasone D (**198**)	Non-withanolidal Steroid	New	*Sinularia brassica*	Inhibited superoxide anion generation and elastase release in fMLP/CB-induced human neutrophils (IC_50_ = 8.4 ± 1.1 and 6.5 ± 1.1 μM) [70]
Flexibilisquinone (**199**)	Quinone derivative	New	*Sinularia flexibilis*	Significantly suppressed the levels of iNOS and COX-2 at 5–20 µM and 20 µM, respectively [71]
Sarcotenuhydroquinone (**200**)	1,4-dihydrobenzoquinone	New	*Sarcophyton tenuispiculatum*	Exhibited cytotoxicity toward MCF-7 and MDA-MB-231 cells (IC_50_ = 25.3 ± 2.8 and 36.4 ± 3.6 μM, respectively) [9]
Crassumtocopherol C (**201**)	α-tocopherol derivative	New	*Lobophytum crassum*	Exhibited cytotoxicity against K562 and Sup-T1 cells (IC_50_ = 34.0 and 23.3 μM, respectively) [7]

## Data Availability

Not applicable.
